# Automated calibration for stability selection in penalised regression and graphical models

**DOI:** 10.1093/jrsssc/qlad058

**Published:** 2023-07-13

**Authors:** Barbara Bodinier, Sarah Filippi, Therese Haugdahl Nøst, Julien Chiquet, Marc Chadeau-Hyam

**Affiliations:** Department of Epidemiology and Biostatistics, MRC Centre for Environment and Health, School of Public Health, Imperial College London, London, UK; Department of Mathematics, Imperial College London, London, UK; Department of Community Medicine, Faculty of Health Sciences, UiT, The Arctic University of Norway, NO-9037 Tromsø, Norway; Université Paris-Saclay, AgroParisTech INRAE, UMR MIA, SolsTIS team, Paris, France; Department of Epidemiology and Biostatistics, MRC Centre for Environment and Health, School of Public Health, Imperial College London, London, UK

**Keywords:** calibration, graphical model, OMICs integration, penalised model, stability selection

## Abstract

Stability selection represents an attractive approach to identify sparse sets of features jointly associated with an outcome in high-dimensional contexts. We introduce an automated calibration procedure via maximisation of an in-house stability score and accommodating a priori-known block structure (e.g. multi-OMIC) data. It applies to [Least Absolute Shrinkage Selection Operator (LASSO)] penalised regression and graphical models. Simulations show our approach outperforms non-stability-based and stability selection approaches using the original calibration. Application to multi-block graphical LASSO on real (epigenetic and transcriptomic) data from the Norwegian Women and Cancer study reveals a central/credible and novel cross-OMIC role of LRRN3 in the biological response to smoking. Proposed approaches were implemented in the R package sharp.

## 1 Introduction

Tobacco smoking has long been established as a dangerous exposure causally linked to several severe chronic conditions, including the risk of developing lung cancer (National Center for Chronic Disease Prevention and Health Promotion (US) Office on Smoking and Health, 2014). Nevertheless, the molecular mechanisms triggered and dysregulated by the exposure to tobacco smoking remain poorly understood. Over the past two decades, OMICs technologies have developed as valuable tools to explore molecular alterations due to external stressors or associated with future health conditions (Niedzwiecki et al., 2019).

Univariate analyses of OMICs data have enabled the identification of molecular markers of exposure to tobacco smoking (Huan et al., 2016; Joehanes et al., 2016). Multivariate regression, where all OMICs markers are used as predictors, can be used to avoid the detection of redundant prospective markers of disease risk (Chadeau-Hyam et al., 2013). In particular, variable selection models can identify sparse and non-redundant sets of predictors and have proved useful for signal prioritisation in this context (Vermeulen et al., 2018). Graphical models are particularly relevant to the analysis of biological data, where we expect intricate relationships between molecular markers (Barabási & Oltvai, 2004). In addition, with the emergence of multi-omics datasets, where multiple high-resolution molecular profiles are measured in the same individuals (Guida et al., 2015; Noor et al., 2019), there is a need for efficient multivariate approaches accommodating high-dimensional and heterogeneous data typically exhibiting block-correlation structures. In this article, we propose some methodological developments to identify (i) smoking-related molecular markers of the risk of developing lung cancer using variable selection, and (ii) relationships between multi-OMICs markers of tobacco smoking using graphical models. These applications are conducted on real multi-OMICs data to illustrate the relevance and utility of the proposed approaches.

For variable selection, we consider the Least Absolute Shrinkage Selection Operator (LASSO), which uses the $\ell_{1}$-penalisation of regression coefficients to induce sparsity (Tibshirani, 1996). Extensions of these penalised regression models have been proposed for the estimation of Gaussian graphical models (Friedman et al., 2007; Meinshausen & Bühlmann, 2006). By applying a $\ell_{1}$-penalisation to the precision matrix (as defined by the inverse of the covariance matrix), the graphical LASSO identifies non-zero entries of the partial correlation matrix. The evaluation (and subsequent selection) of pairwise relationships between molecular features in graphical models can guide biological interpretation of the results, under the assumption that statistical correlations reflect molecular interactions (Barabási & Oltvai, 2004; Valcárcel et al., 2011).

We focus in the present article on the calibration of feature selection models, where feature denotes interchangeably a variable (in the context of regression) or an edge (graphical model). We illustrate our approach with regularised models, in which the model size (number of selected features) is controlled by the penalty parameter. The choice of parameter has strong implications on the generated results. Calibration procedures using cross-validation (Friedman et al., 2010; Leng et al., 2006) or maximisation of information theory metrics, including the Bayesian (BIC) or Akaike (AIC) Information Criterion (Akaike, 1998; Foygel & Drton, 2010; Giraud, 2008; Schwarz, 1978) have been proposed.

These models can be complemented by stability approaches to enhance the reliability of the findings (Liu et al., 2010; Meinshausen & Bühlmann, 2010; Shah & Samworth, 2013). In stability selection, the selection algorithm is combined with resampling techniques to identify the most stable signals. The model relies on the introduction of a second parameter: a threshold in selection proportion above which the corresponding feature is considered stable. A formula providing the upper-bound of the expected number of falsely selected features, or Per-Family Error Rate (PFER), as a function of the two parameters has been derived and is currently used to guide calibration (Meinshausen & Bühlmann, 2010; Shah & Samworth, 2013). However, this calibration relies on the arbitrary choice of one of the two parameters, which can sometimes be difficult to justify.

We introduce a score measuring the overall stability of the set of selected features, and use it to propose a new calibration strategy for stability selection. Our intuition is that all features would have the same probability of being selected in an unstable model. Our calibration procedure does not rely on the arbitrary choice of any parameter. Optionally, the problem can be constrained on the expected number of falsely selected variables and generate sparser results with error control.

We also extend our calibration procedure to accommodate multiple blocks of data. This extension was motivated by the practical example on integration of data from different OMICs platforms. In this setting, block patterns arise, typically with higher (partial) correlations within a platform than between (Canzler et al., 2020). We propose here an extension of stability selection combined with the graphical LASSO accommodating data with a known block structure. For this approach, each block is tuned using a block-specific pair of parameters (penalty and selection proportion threshold) (Ambroise et al., 2009).

We conduct an extensive simulation study to evaluate the performances of our calibrated stability selection models and compare them to state-of-the-art approaches. Our stability selection approaches are applied to targeted methylation and gene expression data from an existing cohort. These datasets are integrated in order to characterise the molecular response to tobacco smoking at multiple molecular levels. The transcript of the LRRN3 gene, and its closest CpG site were found to play a central role in the generated graph. These two variables have the largest numbers of cross-OMICs edges and appear to be linking two largely uni-OMICs modules. LRRN3 methylation and gene expression therefore appear as pivotal molecular signals driving the biological response to tobacco smoking.

## 2 Methods

### 2.1 Data overview

We used DNA methylation and gene expression data in plasma samples from 251 women from the Norwegian Women and Cancer (NOWAC) cohort study (Sandanger et al., 2018). Our study population includes 125 future cases (mean time-to-diagnosis of 4 years) and 126 healthy controls. The data was pre-processed as described elsewhere (Guida et al., 2015). DNA methylation at each CpG site are originally expressed as a proportion of methylated sequences across all copies (*β*-values) and was subsequent ${\text{logit}}_{2}$-transformed (*M*-values). The gene expression data were log-transformed. Features missing in more than 30% of the samples were excluded, and the remaining data was imputed using the k-nearest neighbour. To remove technical confounding, the data was de-noised by extracting the residuals from linear mixed models with the OMIC feature as the outcome and modelling technical covariates (chip and position) as random intercepts (Sandanger et al., 2018).

### 2.2 Motivating research questions

Our overarching research question is to identify the role of smoking-related CpG sites in lung carcingenesis and to better understand the molecular response to the exposure to tobacco smoke.

We therefore identified a subset of 160 CpG sites found differentially methylated in never vs. former smokers at a 0.05 Bonferroni corrected significance level in a large meta-analysis of 15,907 participants from 16 different cohorts (Joehanes et al., 2016). Similarly, we selected a set of 156 transcripts found differentially expressed in never vs. current smokers from a meta-analysis including 10,233 participants from six cohorts (Huan et al., 2016). Of these, 159 CpG sites and 142 transcripts were assayed in our dataset.

Using a logistic-LASSO, we first sought for a sparse subset of the (N=159) assayed smoking-related CpG sites that were jointly associated with the risk of future lung cancer. Second, to characterise the multi-OMICs response to exposure to tobacco smoking we estimated the conditional independence structure between smoking-related CpG sites (N=159) and transcripts (N=142) using the graphical LASSO.

To improve the reliability of our findings, both regularised regression and graphical models are used in a stability selection framework. These analyses raised two statistical challenges regarding the calibration of hyper-parameters in stability selection, and the integration of heterogeneous groups of variables in a graphical model. We detail below our approaches to accommodate these challenges.

### 2.3 Variable selection with the LASSO

In LASSO regression, the $\ell_{1}$-penalisation is used to shrink the coefficients of variables that are not relevant in association with the outcome to zero (Tibshirani, 1996). Let *p* denote the number of variables and *n* the number of observations. Let *Y* be the vector of outcomes of length *n*, and *X* be matrix of predictors of size (n×p). The objective of the problem is to estimate the vector $\beta_{\lambda }$ containing the *p* regression coefficients. The optimisation problem of the LASSO can be written:


(1) $$\min_{\beta_{\lambda }}\sum_{i=1}^{n}(y_{i}-\beta_{\lambda }^{T}x_{i}{)}^{2}+\lambda \sum_{\;j=1}^{p}|\beta_{\lambda_{j}}|,$$

where *λ* is a penalty parameter controlling the amount of shrinkage.

Penalised extensions of models including logistic, Poisson and Cox regressions have been proposed (Simon et al., 2011). In this article, the use of our method is illustrated with LASSO-regularised linear regression. We use its implementation in the glmnet package in R (Gaussian family of models) (Friedman et al., 2010).

### 2.4 Graphical model estimation with the graphical LASSO

A graph is characterised by a set of nodes (variables) and edges (pairwise links between them). As our data are cross-sectional, we focus here on undirected graphs without self-loops. As a result, the adjacency matrix encoding the network structure will be symmetric with zeros on the diagonal.

We assume that the data follows a multivariate Normal distribution:


(2) $$X_{i}∼N_{p}(\mu ,\Sigma ),\;i\in \{1,\ldots ,n\},$$

where *μ* is the mean vector and Σ is the covariance matrix.

The conditional independence structure is encoded in the support of the precision matrix $\Omega =\Sigma^{-1}$. Various extensions of the LASSO have been proposed for the estimation of a sparse precision matrix (Banerjee et al., 2008; Meinshausen & Bühlmann, 2006). We use here the graphical LASSO (Friedman et al., 2007) as implemented in the glassoFast package in R (Friedman et al., 2018; Sustik & Calderhead, 2012; Witten et al., 2011). For a given value of the penalty parameter *λ*, the optimisation problem can be written as:


(3) $$\max_{\Omega }\;\text{log}\;\text{det}\;(\Omega )-\text{tr}\;(S\Omega )-\lambda \|\Omega {\|}_{\ell_{1}},$$

where *S* is the empirical covariance matrix and $\left\|\Omega {\|}_{\ell_{1}}=\sum_{i\neq j}|\Omega_{ij}\right|$.

Alternatively, a penalty matrix Λ can be used instead of the scalar *λ* for more flexible penalisation:


(4) $$\max_{\Omega }\;\text{log}\;\text{det}\;(\Omega )-\text{tr}\;(S\Omega )-\|\Lambda \;∙\;\Omega {\|}_{\ell_{1}},$$

where ∙ denotes the element-wise matrix product.

### 2.5 Stability selection

Stability-enhanced procedures for feature selection proposed in the literature include stability selection (Meinshausen & Bühlmann, 2010; Shah & Samworth, 2013) and the Stability Approach to Regularization Selection (StARS) (Liu et al., 2010). Both use an existing selection algorithm and complement it with resampling techniques to estimate the probability of selection of each feature using its selection proportion over the resampling iterations. Stability selection ensures reliability of the findings through error control.

The feature selection algorithms we use are (a) the LASSO in a regression framework (Friedman et al., 2010; Tibshirani, 1996) and (b) the graphical LASSO for the estimation of Gaussian graphical models (Banerjee et al., 2008; Meinshausen & Bühlmann, 2006; Sustik & Calderhead, 2012) (see Supplementary Methods, Sections 1.1 and 1.2 for more details on the algorithms). The latter aims at the construction of a conditional independence graph. In a graph with *p* nodes, for each pair of variables X,Y and Gaussian vector *Z* compiling the (p−2) other variables, an edge is included if the conditional covariance cov(X,Y∣Z) is different from zero (see Supplementary Methods, Section 1.3 for more details on model calibration).

Under the assumption that the selection of feature *j* is independent from the selection of any other feature i≠j, the binary selection status of feature *j* follows a Bernouilli distribution with parameter $p_{\lambda }(j)$, the selection probability of feature *j*. The stability selection model is then defined as the set $V_{\lambda ,\pi }$ of features with selection probability above a threshold *π*:


(5) $$V_{\lambda ,\pi }=\{j\;:\;p_{\lambda }(j)\geq \pi \}.$$

For each feature *j*, the selection probability is estimated as the selection proportion across models with penalty parameter *λ* applied on *K* subsamples of the data.

The stability selection model has two parameters (λ,π) that need to be calibrated. In the original paper, Meinshausen & Bühlmann use random subsamples of 50% of the observations. They introduce $q_{\Lambda }$, the average number of features that are selected at least once by the underlying algorithm (e.g. LASSO) for a range of values λ∈Λ, across the *K* subsamples. Under the assumptions of (a) exchangeability between selected features and (b) that the selection algorithm is not performing worse than random guessing, they derived an upper-bound of the PFER, denoted by ${\text{PFER}}_{\mathrm{MB}}$, as a function of the number of selected features $q_{\Lambda }$ and threshold in selection proportion *π*:


$${\text{PFER}}_{\mathrm{MB}}(\Lambda ,\pi )=\frac{1}{2\pi -1}\frac{q_{\Lambda }^{2}}{N}.$$

With simultaneous selection in complementary pairs (CPSS), the selection proportions are obtained by counting the number of times the feature is selected in both the models fitted on a subsample of 50% of the observations and its complementary subsample made of the remaining 50% of observations (Shah & Samworth, 2013). Using this subsampling procedure, the exchangeability assumption is not required for the upper-bound ${\text{PFER}}_{\mathrm{MB}}$ to be valid. Under the assumption of unimodality of the distribution of selection proportions obtained with CPSS, Shah & Samworth also proposed a stricter upper-bound on the expected number of variables with low selection probabilities, denoted here by ${\text{PFER}}_{\mathrm{SS}}$:


$${\text{PFER}}_{\mathrm{SS}}(\Lambda ,\pi )=\left\{ \begin{array}{cc} \frac{1}{2\times (2\pi -1-1/K)}\frac{q_{\Lambda }^{2}}{N} & \text{if}\;\pi \leq 0.75\\ \frac{4\times (1-\pi +1/K)}{1+2/K}\frac{q_{\Lambda }^{2}}{N} & \text{otherwise.} \end{array}\right.$$

For simplicity, we consider here point-wise control (Λ reduces to a single value *λ*) with no effects on the validity of the formulas. Both approaches provide a relationship between *λ* (via $q_{\lambda }$), *π* and the upper-bound of the PFER such that if two of them are fixed, the third one can be calculated. The authors of both papers proposed to guide calibration based on the arbitrary choice of two of these three quantities. For example, the penalty parameter *λ* can be calibrated for a combination of fixed values of the selection proportion *π* and threshold in PFER.

To avoid the arbitrary choice of the selection proportion *π* or penalty *λ*, we introduce here a score measuring the overall stability of the model and use it to jointly calibrate these two parameters. We also consider the use of a user-defined threshold in PFER to limit the set of parameter values for *λ* and *π* to explore.

### 2.6 Stability score

Our calibration procedure aims at identifying the pair of hyper-parameters (λ,π) that maximises model stability (Yu, 2013). Let $H_{\lambda }(j)\in \{0,\ldots ,K\}$ denote the selection count of feature j∈{1,…,N} calculated over the *K* models fitted with parameter *λ* over different subsamples. To quantify model stability, we first define three categories of features based on their selection counts. For a given penalty parameter *λ* and threshold in selection proportion π∈]0.5,1[, each feature *j* is either (a) stably selected if $H_{\lambda }(j)\geq K\pi$, (b) stably excluded if $H_{\lambda }(j)\leq K(1-\pi )$, or (c) unstably selected if $(1-\pi )K<H_{\lambda }(j)<K\pi$. Unstably selected features are those that are ambiguously selected across subsamples. The partitioning of the features into these three categories provides information about model stability, whereby a stable model would include a large numbers of stably selected and/or stably excluded features and a small number of unstably selected features.

We hypothesise that under the most unstable selection procedure, all features would have the same probability $\gamma_{\lambda }=q_{\lambda }/N$ of being selected, where $q_{\lambda }=\lfloor \frac{1}{K}\sum_{\;j=1}^{N}H_{\lambda }(j)+\frac{1}{2}\rfloor$ is the average number of selected features across the *K* models fitted with penalty *λ* on the different subsamples of the data. Further assuming that the subsamples are independent, the selection count $H_{\lambda }(j)$ of feature j∈{1,…,N} would then follow a binomial distribution:


$$H_{\lambda }(j)∼B(K,\gamma_{\lambda }).$$

By considering the *N* selection counts as independent observations, we can derive the likelihood of observing this classification under the hypothesis of instability, given *λ* and *π*:


$$\begin{array}{rl} L_{\lambda ,\pi } & =\prod_{\;j=1}^{N}[{\left(1-F_{K,\gamma_{\lambda }}(K\pi -1)\right)}^{1_{\left\{H_{\lambda }(j)\geq K\pi \right\}}}\\ & \;\times {\left(F_{K,\gamma_{\lambda }}(K\pi -1)-F_{K,\gamma_{\lambda }}(K(1-\pi ))\right)}^{1_{\left\{(1-\pi )K<H_{\lambda }(j)<K\pi \right\}}}\\ & \;\;\times \;F_{K,\gamma_{\lambda }}(K(1-\pi ){)}^{1_{\left\{H_{\lambda }(j)\leq K(1-\pi ),\right\}}}], \end{array}$$

where $F_{K,\gamma_{\lambda }}$ is the cumulative probability function of the binomial distribution with parameters *K* and $\gamma_{\lambda }$.

Our stability score $S_{\lambda ,\pi }$ is defined as the negative log-likelihood under the hypothesis of equi-probability of selection:


$$S_{\lambda ,\pi }=-\text{log}\;(L_{\lambda ,\pi }).$$

The score $S_{\lambda ,\pi }$ measures how unlikely a given model is to arise from the null hypothesis, for a given set of *λ* and *π*. As such, the higher the score, the more stable the set of selected features. By construction, this formula is accounting for (a) the total number of features *N*, (b) the number of iterations *K*, (c) the density of selected sets by the original procedure via *λ*, and (d) the level of stringency as measured by threshold *π*. The calibration approach we develop aims at identifying sets of parameters *λ* and *π* maximising our score:


(6) $$\max_{\lambda ,\pi }\;S_{\lambda ,\pi }.$$

Furthermore, this calibration technique can be extended to incorporate some error control via a constraint ensuring that the expected number of false positives (FP) is below an a priori fixed threshold in PFER *η*:


(7) $$\max_{\lambda ,\pi }\;S_{\lambda ,\pi }\;\text{such that}\;U_{\lambda ,\pi }\leq \eta ,\text{where}$$

$U_{\lambda ,\pi }$ is the upper-bound used for error control in existing strategies (i.e. ${\text{PFER}}_{\mathrm{MB}}$ or ${\text{PFER}}_{\mathrm{SS}}$) (Meinshausen & Bühlmann, 2010; Shah & Samworth, 2013).

In the following sections, the use of equation (6) is referred to as unconstrained calibration, and that of equation (7) as constrained calibration.

### 2.7 Multi-block graphical models

The combination of heterogeneous groups of variables can create technically induced patterns in the estimated (partial) correlation matrix, subsequently inducing bias in the generated graphical models. This can be observed, for example, when integrating the measured levels of features from different OMICs platforms. The between-platform (partial) correlations are overall weaker than within platforms (Supplementary Material, Figure S1). This makes the detection of bipartite edges more difficult. This structure is known a priori and does not need to be inferred from the data. Indeed, the integration of data arising from *G* homogeneous groups of variables generates $B=\frac{G\times (G+1)}{2}$ two-dimensional blocks in the (partial) correlation matrix where variables are ordered by group (Ambroise et al., 2009).

To tackle this scaling issue, we propose to use and calibrate block-specific pairs of parameters, $\lambda_{b}$ and $\pi_{b}$ controlling the level of sparsity in block *b*. Let $E_{b},b\in \{1,\ldots ,B\}$ denote the sets of edges belonging to each of the blocks, such that:


$$\overset{B}{\underset{b=1}{⋃}}E_{b}=\{1,\ldots ,N\}.$$

The stability selection model can be defined more generally as:


(8) $$V_{\lambda_{1},\ldots ,\lambda_{B},\pi_{1},\ldots ,\pi_{B}}=\overset{B}{\underset{b=1}{⋃}}\{j\in E_{b}\;:\;p_{\lambda_{1},\ldots ,\lambda_{B}}(j)\geq \pi_{b}\},\;\text{where}$$

The probabilities $p_{\lambda_{1},\ldots ,\lambda_{B}}(j),j\in \{1,\ldots ,N\}$ are estimated as selection proportions of the edges obtained from graphical LASSO models fitted on *K* subsamples of the data with a block penalty matrix such that edge $j\in E_{b}$ is penalised with $\lambda_{b}$.

Our stability score is then defined, by block, as:


$$\begin{array}{rl} S_{\lambda_{1},\ldots ,\lambda_{B},\pi_{1},\ldots ,\pi_{B}} & =-\log\;(\prod_{b=1}^{B}\prod_{\;j\in E_{b}}[{\left(1-F_{K,\gamma_{\lambda_{1},\ldots ,\lambda_{B}}}(K\pi_{b}-1)\right)}^{1_{\left\{H_{\lambda_{1},\ldots ,\lambda_{B}}^{b}(j)\geq K\pi_{b}\right\}}}\\ & \;\;\;\times {\left(F_{K,\gamma_{\lambda_{1},\ldots ,\lambda_{B}}}(K\pi_{b}-1)-F_{K,\gamma_{\lambda_{1},\ldots ,\lambda_{B}}}(K(1-\pi_{b}))\right)}^{1_{\left\{(1-\pi_{b})K<H_{\lambda_{1},\ldots ,\lambda_{B}}^{b}(j)<K\pi_{b}\right\}}}\\ & \;\;\;\times F_{K,\gamma_{\lambda_{1},\ldots ,\lambda_{B}}}(K(1-\pi_{b}){)}^{1_{\left\{H_{\lambda_{1},\ldots ,\lambda_{B}}^{b}(j)\leq K(1-\pi_{b})\right\}}}]). \end{array}$$

Alternatively, we propose a block-wise decomposition, as described in equation (9). To ensure that pairwise partial correlations in each block are estimated conditionally on all other (p−2) nodes, we propose to estimate them from graphical LASSO models where the other blocks are weakly penalised (i.e. with small penalty $\lambda_{0}$). We introduce $p_{\lambda_{b},\lambda_{0}}^{b}(j)$ and $H_{\lambda_{b},\lambda_{0}}^{b}(j)$, the selection probability and count of edge $j\in E_{b}$ as obtained from graphical LASSO models fitted with a block penalty matrix such that edges $j\in E_{b}$ are penalised with $\lambda_{b}$ and edges $i\in E_{\ell },\ell \neq b$ are penalised with $\lambda_{0}$. We define the multi-block stability selection graphical model as the union of the sets of block-specific stable edges:


(9) $$V_{\lambda_{1},\ldots ,\lambda_{B},\lambda_{0},\pi_{1},\ldots ,\pi_{B}}=\overset{B}{\underset{b=1}{⋃}}\{j\in E_{b}\;:\;p_{\lambda_{b},\lambda_{0}}^{b}(j)\geq \pi_{b}\}.$$

The pair of parameters is calibrated for each of the blocks separately using a block-specific stability score defined by:


$$\begin{array}{rl} S_{\lambda_{b},\lambda_{0},\pi_{b}}^{b} & =-\log\;(\prod_{\;j\in E_{b}}[{\left(1-F_{K,\gamma_{\lambda_{b},\lambda_{0}}}(K\pi_{b}-1)\right)}^{1_{\left\{H_{\lambda_{b},\lambda_{0}}^{b}(j)\geq K\pi_{b}\right\}}}\\ & \;\;\times {\left(F_{K,\gamma_{\lambda_{b},\lambda_{0}}}(K\pi_{b}-1)-F_{K,\gamma_{\lambda_{b},\lambda_{0}}}(K(1-\pi_{b}))\right)}^{1_{\left\{(1-\pi_{b})K<H_{\lambda_{b},\lambda_{0}}^{b}(j)<K\pi_{b}\right\}}}\\ & \;\;\times \;F_{K,\gamma_{\lambda_{b},\lambda_{0}}}(K(1-\pi_{b}){)}^{1_{\left\{H_{\lambda_{b},\lambda_{0}}^{b}(j)\leq K(1-\pi_{b})\right\}}}]), \end{array}$$

where $\gamma_{\lambda_{b},\lambda_{0}}$ is calculated based on the selection counts in $H_{\lambda_{b},\lambda_{0}}^{b}$.

The implication of these assumptions are evaluated by comparing the two approaches described in equations (9) and (8) in a simulation study.

### 2.8 Implementation

The stability selection procedure is applied for different values of *λ* and *π* and the stability score is computed for all visited pairs of parameters. The grid of *λ* values is chosen so that the underlying selection algorithm visits a range of models from empty to dense (up to 50% of edges selected by the graphical LASSO) (Friedman et al., 2010; Müller et al., 2016). Values of the threshold *π* vary between 0.6 and 0.9, as proposed previously (Meinshausen & Bühlmann, 2010).

### 2.9 Simulation models

In order to evaluate the performances of our approach and compare to other established calibration procedures, we simulated several datasets according to the models described below, which we implemented in the R package fake (version 1.3.0).

#### 2.9.1 Graphical models

We build upon previously proposed models to simulate multivariate Normal data with an underlying graph structure (Zhao et al., 2012). Our contributions include (a) a procedure for the automated choice of the parameter ensuring that the generated correlation matrix has contrast, and (b) the simulation of block-structured data.

First, we simulate the binary adjacency matrix Θ of size (p×p) of a random graph with density *ν* using the Erdös–Rényi model (Erdös & Rényi, 1959) or a scale-free graph using the Barabási-Albert preferential attachment algorithm (Albert & Barabási, 2002; Zhao et al., 2012). To introduce a block structure in the generate data, the non-diagonal entries of the precision matrix Ω are simulated such that:


$$\Omega_{ij}=\left\{ \begin{array}{cc} \;\;\;\;\;\;\;\;0 & \;\text{if}\;\Theta_{ij}=0\\ \;\;\;\;\;\;\alpha_{ij} & \;\;\;\;\text{if}\;\Theta_{ij}=1\;\text{and}\;i\;\text{and}\;j\;\text{belong to the same platform}\\ \alpha_{ij}v_{b} & \text{if}\;\Theta_{ij}=1\;\text{and}\;i\;\text{and}\;j\;\text{belong to different platforms.} \end{array}\right.,\;i\neq j\;\text{where}$$

$\alpha_{ij}∼U(\{-1,1\})$ and $v_{b}\in [0,1]$ is a user-defined parameter.

We ensure that the generated precision matrix is positive definite via diagonal dominance:


$$\Omega_{ii}=\sum_{\;j=1}^{p}|\Omega_{ij}|+u,\;\text{where}$$

u>0 is a parameter to be tuned.

The data are simulated from the centred multivariate Normal distribution with covariance $\Omega^{-1}$.

The simulation model is controlled by five parameters:

- number of observations *n*,
- number of nodes *p*,
- density of the underlying graph ν∈[0,1],
- scaling factor $v_{b}\in [0,1]$ controlling the level of heterogeneity between blocks,
- constant u>0 ensuring positive definiteness.

We propose to choose *u* so that the generated correlation matrix has a high contrast, as defined by the number of unique truncated correlation coefficients with three digits (Supplementary Material, Figure S2). The parameter $v_{b}\in [0,1]$ is set to 1 (no block structure) for single-block simulations and chosen to generate data with a visible block structure for multi-block simulations ($v_{b}=0.2$). These models generate realistic correlation matrices (Supplementary Material, Figure S1).

#### 2.9.2 Linear regression

For linear regression, the data simulation is done in two steps with (i) the simulation of *n* observations for the *p* predictors and (ii) the simulation of the outcome for each of the *n* observations, conditionally on the predictors data. The first step is done using the simulation model introduced in the previous section for graphical models. This allows for some flexibility over the (conditional) independence patterns between predictors. For the second step, we sample *β*-coefficients from a uniform distribution over {−1,1} (for homogeneous effects in absolute value) or over {[−1,0.5]∪[0.5,1]} (to introduce variability in the strength of association with the outcome). The outcome $Y_{i},i\in \{1,\ldots ,n\}$ is then sampled from a Normal distribution (Friedman et al., 2010):


$$Y_{i}|X_{i}=x_{i}∼N(x_{i}\beta ,\sigma^{2}).$$

The parameter *σ* controls the proportion of variance in the outcome that can be explained by its predictors. The value of *σ* is chosen to reach the expected proportion of explained variance $R^{2}$ used as simulation parameter:


$$\sigma =\sqrt{\frac{1-R^{2}}{R^{2}}s^{2}},$$

where $s^{2}$ is the variance of Xβ.

#### 2.9.3 Performance metrics

Selection performances of the investigated models are measured in terms of precision *p* and recall *r*:


$$p=\frac{\mathrm{TP}}{\mathrm{TP}+\mathrm{FP}}\;\text{and}\;r=\frac{\mathrm{TP}}{\mathrm{TP}+\mathrm{FN}},\;\text{where}$$

TP and FP are the numbers of true and false positives, respectively, and FN is the number of FNs.

The $F_{1}$-score quantifies the overall selection performance based on a single metric:


$$F_{1}=\frac{2\times p\times r}{p+r}$$

## 3 Simulation study

We use a simulation study to demonstrate the relevance of stability selection calibrated with our approach:

- in a linear regression context for the LASSO model,
- for graphical model using the graphical LASSO,
- for multi-block graphical models.

From these, we evaluate the relevance of our stability score for calibration purposes, and compare our score to a range of existing calibration approaches including information theory criteria, StARS, and stability selection models using the previously proposed error control for different values of the threshold in selection proportion *π*. As sensitivity analyses, we evaluate the performances of stability selection for graphical models using different resampling approaches, different numbers of iterations *K*, and compare the two proposed approaches for multi-block calibration.

### 3.1 Simulation parameters

All simulation parameters were chosen in an attempt to generate realistic data with many strong signals and some more difficult to detect (weaker partial correlation).

For graphical models, we used p=100 nodes with an underlying random graph structure of density ν=0.02 (99 edges on average, as would be obtained in a scale-free graph with the same number of nodes). For multi-block graphical models, we considered two homogeneous groups of 50 nodes each. Reported distributions of selection metrics were computed over 1,000 simulated datasets.

Unless otherwise stated, stability selection models were applied on grids of 50 dataset-specific penalty parameter values and 31 values for the threshold in selection proportion between 0.6 and 0.9. The stability-enhanced models were based on K=100 (complementary) subsamples of 50% of the observations.

### 3.2 Applications to simulated data

**Figure 1.**
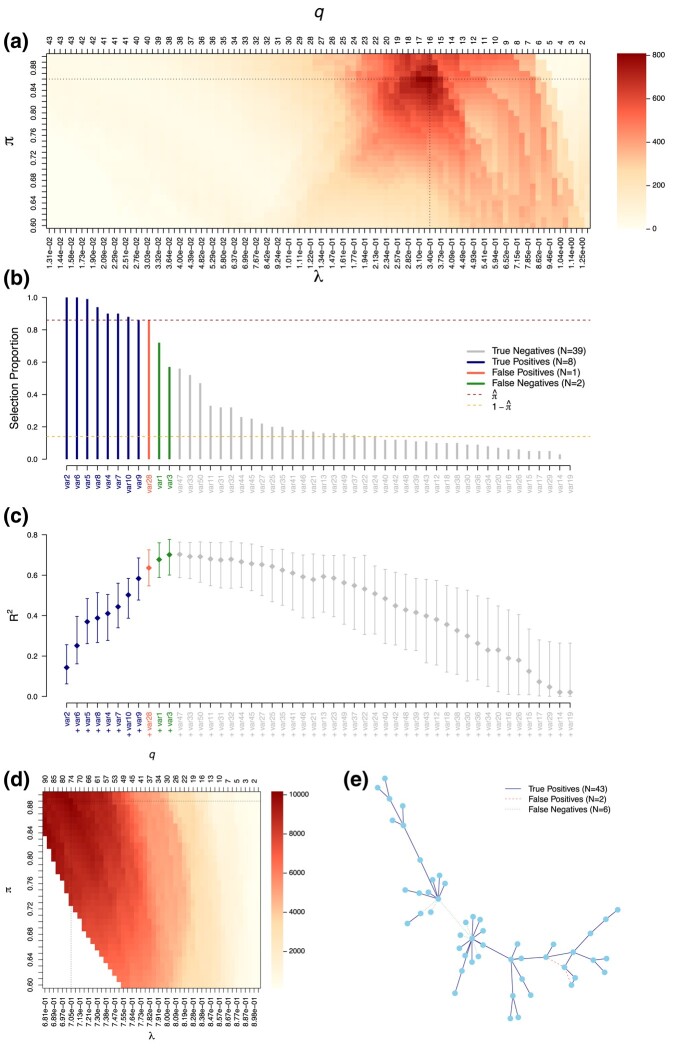
Stability selection LASSO (a–c) and graphical LASSO (D–E) applied on simulated data. Calibration plots (a–d) show the stability score (colour-coded) for different penalty parameters *λ*, or numbers of features selected *q*, and thresholds in selection proportion *π*. We show selection proportions (b) and a graph representation of the detected and missed edges (e). We report the median, 5th and 95th quantiles of the $R^{2}$ obtained for 100 unpenalised regression models sequentially adding the predictors in order of decreasing selection proportions (c). These models are trained on 50% of the data and performances are evaluated on the remaining observations. TPs, FPs, and FN are highlighted (b, c, e). Calibration of the stability selection graphical LASSO ensures that the expected number of false positives (PFER) is below 20 (d). The two datasets are simulated for p=50 variables and n=100 observations. For the regression model, 10 variables contribute to the definition of the outcome with effect sizes in {[−1,−0.5]∪[0.5,1]} and an expected proportion of explained variance of 70%. For the graphical model, the simulated graph is scale-free.

Our stability selection approach is first applied to the LASSO for the selection of variables jointly associated with a continuous outcome in simulated data (Figure 1).

The penalty parameter *λ* and threshold in selection proportion *π* are jointly calibrated to maximise the stability score (Figure 1a). Stably selected variables are then identified as those with selection proportions greater than the calibrated parameter $\hat{\pi }=0.86$ (dark red line) in LASSO models fitted on 50% of the data with calibrated penalty parameter $\hat{\lambda }=0.34$ (Figure 1b). The resulting set of stably selected variables includes 8 of the 10 ‘true’ variables used to simulate the outcome and 1 ‘wrongly selected’ variables we did not use in our simulation.

We observe a marginal increase in prediction performances across unpenalised models sequentially adding the nine stably selected predictors by order of decreasing selection proportions (Figure 1c). Further including the two FNs generates a limited increase in $R^{2}$, and so does the inclusion of any subsequent variable. This suggests that our stability selection model captures most of the explanatory information and was therefore well calibrated.

To limit the number of ‘wrongly selected’ features, we can restrict the values of *λ* and *π* visited so they ensure a control of the PFER (Supplementary Material, Figure S3). In that constrained optimisation, the values of *λ* and *π* yielding a PFER exceeding the specified threshold are discarded and corresponding models are not evaluated (Supplementary Material, Figure S3a). The maximum stability score can be obtained for different pairs (λ,π) depending on the constraint, but our simulation shows that differences in the maximal stability score (Supplementary Material, Figure S3b) and resulting selected variables are small (Supplementary Material, Figure S3c) if the constraint is not too stringent.

Our stability score is also used to calibrate the graphical LASSO for the estimation of a conditional independence graph, while controlling the expected number of falsely selected edges below 20 (Figure 1c). The calibrated graph (Figure 1d) included 56 (47 rightly, in plain dark blue and 9 wrongly, in dashed red lines) stably selected edges (i.e. with selection proportions $\geq \hat{\pi }=0.90$), based on graphical LASSO models fitted on 50% of the data with penalty parameter λ=0.52. The nine wrongly selected edges tend to be between nodes that are otherwise connected in this example (marginal links). The two missed edges are connected to the central hub and thus correspond to smaller partial correlations, more difficult to detect.

### 3.3 Evaluation of model performance and comparison with existing approaches

**Figure 2.**
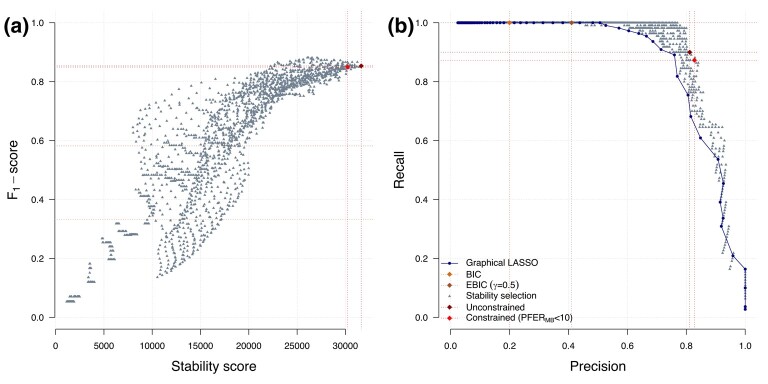
Selection performance in stability selection and relevance of the stability score for calibration. The graphical LASSO and stability selection are applied on simulated data with n=200 observations for p=100 variables where the conditional independence structure is that of a random network with ν=0.02. The $F_{1}$-score of stability selection models fitted with a range of *λ* and *π* values is represented as a function of the stability score (a). Calibrated stability selection models using the unconstrained and constrained approaches are highlighted. The precision and recall of visited stability selection models (grey) and corresponding graphical LASSO models (dark blue) are reported (b). The calibrated models using the BIC (beige) or EBIC (brown) are also showed (b).

Our simulations show that models with higher stability score yield higher selection performances (as measured by the $F_{1}$-score), making it a relevant metric for calibration (Figure 2a). We also find that irrespective of the value of *λ* and *π*, stability selection models outperform the original implementation of the graphical LASSO (Figure 2b). Graphical LASSO calibrated using the BIC or EBIC (see Supplementary Methods, Supplementary Material, Section 1.3) generate dense graphs resulting in perfect recall and poor precision values (0.20 and 0.41). Our stability score instead yield sparser models, resulting in slightly lower recall values (0.90) which did not include many irrelevant edges, as captured by the far better precision value (0.81). Our simulation also shows that the constraint controlling the PFER further improves the precision (0.83) through the generation of a sparser model.

**Figure 3.**
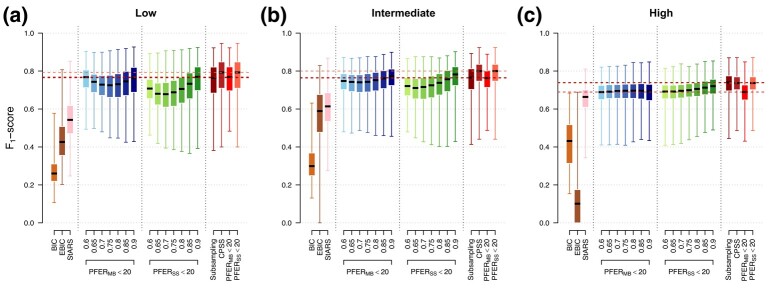
Selection performances of state-of-the-art approaches and proposed calibrated stability selection graphical LASSO models. We show the median, quartiles, minimum, and maximum $F_{1}$-score of graphical LASSO models calibrated using the BIC, EBIC, StARS, and stability selection graphical LASSO models calibrated via error control (MB or SS) or using the proposed stability score (red). Models are applied on 1,000 simulated datasets with p=100 variables following a multivariate Normal distribution corresponding to a random graph structure (ν=0.02). Performances are estimated in low (n=2p=200, a), intermediate (n=p=100, b), and high (n=p/2=50, c) dimensions.

Our calibrated stability selection graphical LASSO models are compared with state-of-the-art graphical model estimation approaches on 1,000 simulated datasets in low, intermediate and high-dimension (Figure 3, Supplementary Material, Table S1). Non stability-enhanced graphical LASSO models, calibrated using information theory criteria, are generally the worst performing models (median $F_{1}$-score <0.6 across dimensionality settings). StARS models, applied with the same number of subsampling iterations and using default values for other parameters, have the highest median numbers of TPs. However, they include more FPs than stability selection models, making it less competitive in terms of $F_{1}$-score (best performing in high-dimension with a median $F_{1}$-score of 0.66). For stability selection models calibrated using error control (MB Meinshausen & Bühlmann, 2010, SS Shah & Samworth, 2013), the optimal choice of *π* seems to depend on many parameters including the dimensionality and structure of the graph (Supplementary Material, Figure S4). By jointly calibrating the two parameters, our models show generally better performances compared to models calibrated solely using error control on these simulations (median $F_{1}$-score ranging from 0.69 to 0.72 using ${\text{PFER}}_{\mathrm{SS}}<20$ only in high dimension, compared to 0.74 using constrained calibration maximising the stability score). Results were consistent when using different thresholds in PFER (Supplementary Material, Figure S5). For LASSO models, we observe a steep increase in precision with all stability selection models compared to models calibrated by cross-validation (Supplementary Material, Figure S6). Unconstrained calibration using our stability score yielded the highest $F_{1}$-scores in the presence of independent or correlated predictors. Computation times of the reported stability selection models are comparable and acceptable in practice (less than 3 min in these settings) but rapidly increase with the number of nodes for graphical models, reaching 8 hr for 500 observations and 1,000 nodes (Supplementary Material, Table S2).

### 3.4 Sensitivity to the choice of resampling procedure

Stability selection can be implemented with different numbers of iterations *K* and resampling techniques (subsampling, bootstrap or CPSS approaches, and subsample size). We show in a simulation study with p=100 nodes that (a) the effect of the number of iterations *K* reaches a plateau after 50 of iterations, and (b) that the best performances were obtained for bootstrap samples or subsamples of 50% of the observations (Supplementary Material, Figures S7 and S8).

### 3.5 Multi-block extension for graphical models

**Figure 4.**
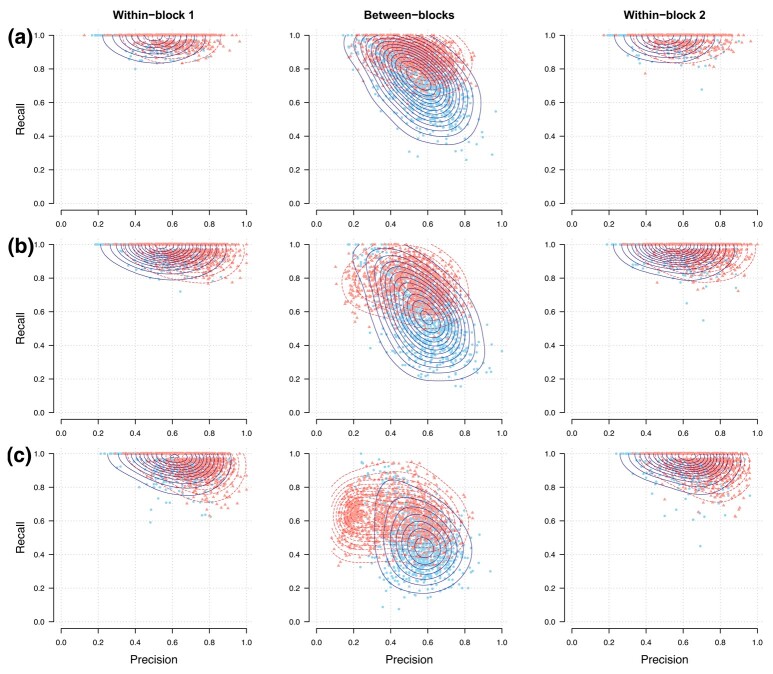
Precision-recall showing single and multi-block stability selection graphical models applied on simulated data with a block structure. Models are applied on 1,000 simulated datasets (points) with p=100 variables following a multivariate Normal distribution corresponding to a random graph (ν=0.02) and with known block structure (50 variables per group, using $v_{b}=0.2$). The contour lines indicate estimated two-dimensional density distributions. Performances are evaluated in low (a, n=2p=200), intermediate (b, n=p=100), and high (c, n=p/2=50) dimensions.

Our single and multi-block calibration procedures are applied on simulated datasets with a block structure in different dimensionality settings. Block-specific selection performances of both approaches can be visualised in precision-recall plots (Figure 4, Supplementary Material, Table S3). Irrespective of the dimensionality, accounting for the block structure as proposed in equation (9) with $\lambda_{0}=0.1$ generates an increase in selection performance in both within and between blocks (up to 7% in overall median $F_{1}$-score in low dimension). This gain in performance comes at the price of an increased computation time (from 2 to 6 min in low dimension).

Additionally, we show in Supplementary Material, Table S4 that the choice of $\lambda_{0}$ has limited effects on the selection performances, as long as it is relatively small ($\lambda_{0}\leq 0.1$). We choose $\lambda_{0}=0.1$ for a good balance between performance and computation time. We also show that the use of equation (9) gives better selection performances than that of equation (8) (median ${\text{F}}_{1}$-score ≥0.71 compared to 0.57). In particular, it drastically reduces the numbers of FPs in the off-diagonal block.

## 4 Application: molecular signature of smoking

### 4.1 Epigenetic markers of lung cancer

**Figure 5.**
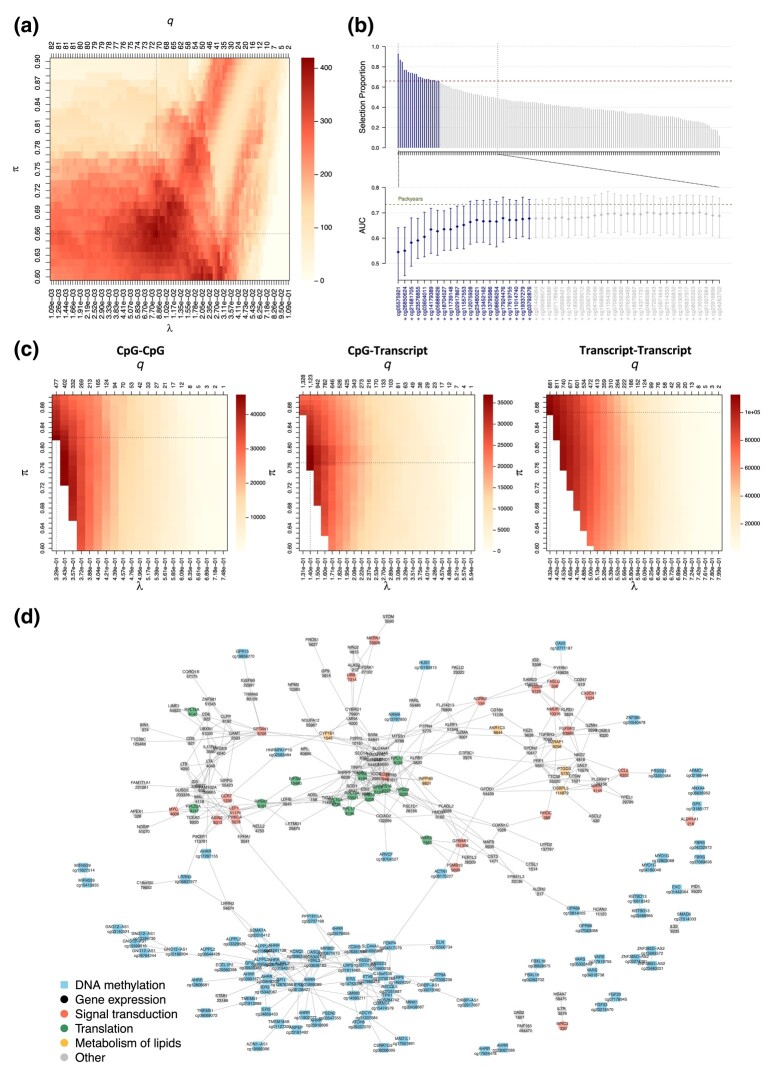
Stability selection on real DNA methylation and gene expression data. The stability selection logistic-LASSO with the future lung cancer status as outcome and epigenetic markers of smoking as predictors is calibrated by maximising the stability score (a). The selection proportions in the calibrated model and explanatory performances of unpenalised logistic models where the predictors are sequentially added by decreasing selection proportion are showed (b). The three blocks of a multi-OMICs graphical model integrating DNA methylation and gene expression markers of tobacco smoking are calibrated separately using models where the other blocks are weakly penalised ($\lambda_{0}=0.1$), while ensuring that ${\text{PFER}}_{\mathrm{MB}}<150$ overall (c). The stability selection model includes edges that are stably selected in each block (d).

To identify smoking-related markers that contribute to the risk of developing lung cancer, we use stability selection logistic-LASSO with the 159 CpG sites as predictors and the future lung cancer status as outcome (Figure 5a, b). The calibrated model includes 21 CpG sites with selection proportions above 0.66. The unpenalised logistic models with stably selected predictors reach a median AUC of 0.69, which is close to that of pack years (median AUC of 0.74) and implies that these 21 CpG sites capture most of the information on smoking history relevant to lung cancer prediction. The limited increase in AUC beyond the calibrated number of predictors suggests that the stability selection model achieves a good balance between sparsity and prediction performance.

### 4.2 Multi-OMICs graph

We first estimate the conditional independence structure between smoking-related CpG sites with single-block stability selection (Supplementary Material, Figure S9). A total of 320 edges involving 100 of the 159 CpG sites are obtained. Most CpG sites are in the same connected component, but we also observe 6 small modules made of 2 or 3 nodes.

In order to get a more comprehensive understanding of the biological response to smoking we integrate methylation data, known to reflect long-term exposure to tobacco smoking, and gene expression, which is functionally well characterised, and seek for correlation patterns across these smoking-related signals via the estimation of a multi-OMICs graph.

We accommodate the heterogeneous data structure (Supplementary Material, Figure S10) by calibrating three pairs of block-specific parameters (λ,π) using our multi-block strategy (Figure 5a). We found a total of 601 edges, including 150 in the within-methylation block, 425 in the within-gene expression block, and 26 cross-OMICs edges (Figure 5b). The detected links reflect potential participation to common regulatory processes of both transcripts and CpG sites. As our analysis was limited to smoking-related markers, connected nodes can be hypothesised to jointly contribute to the biological response to tobacco smoking.

For comparison, we estimate the graphical LASSO model calibrated using the BIC on the same data (Supplementary Material, Figure S11). Of the 601 edges included in the stability selection graph, 583 were also in the BIC-calibrated graph. The BIC-calibrated graph is more dense (N= 6,744 edges), which makes it difficult to interpret. As this procedure does not account for the block structure in the data, two modules corresponding to the two platforms are clearly visible.

DNA methylation nodes are annotated with the symbol of its closest gene on the genome (Joehanes et al., 2016). Most sets of CpG sites annotated with the same gene symbol are interconnected in the graph (e.g. AHRR, GNG12-AS1, and ALPPL2 on chromosomes 5, 3, and 2, respectively). The data includes a CpG site and a transcript with the same annotation for two genes, but only found a cross-OMIC link for LRRN3 (Guida et al., 2015). The LRRN3 transcript, which is linked to 4 CpG sites including AHRR, ALPPL2, and a CpG site annotated as LRRN3 (cg09837977), has a central position among methylation markers (Figure 5b).

Strong correlations involving features that are closely located on the genome, or cis-effects, have been reported previously (Robinson et al., 2014). Our approach also detects cross-chromosome edges (Supplementary Material, Figure S12), suggesting that complex long-range mechanisms could be at stake (Jones, 2012).

We incorporate functional information in the visualisation using Reactome pathways (Figure 5b) (Jassal et al., 2020; Langfelder & Horvath, 2008). As previously reported, the immune system and in signal transduction (red) pathways were largely represented in the targeted set (Huan et al., 2016; Sandanger et al., 2018). Interestingly, the group of interconnected nodes around RPL4 (green) was involved in a range of pathways including the cellular response to stress, translation, and developmental biology. Similarly, the transcripts involved in the metabolism of lipids (yellow) are closely related in the graph. Altogether these results confirm the functional proximity of the nearby variables from our graph, hence lending biological plausibility of its topology.

## 5 Discussion

The stability selection models and proposed calibration procedure have been implemented in the R package sharp (version 1.2.1), available on CRAN. The selection performances of our variable selection and (multi-block) graphical models were evaluated in a simulation study. We showed that stability selection models yield higher ${\text{F}}_{1}$-score, to the cost of a (limited) increase in computation time. The computational efficiency of the proposed approaches can easily be improved using warm start and parallelisation, both readily implemented in the R package sharp. We also demonstrated that the proposed calibration procedure is generally identifying the optimal threshold in selection proportion which leads to overall equivalent or better performances than previously proposed approaches based solely on error control. Our multi-block extension was successful in removing some of the technical bias through a more flexible modelling, but generated a tenfold increase in computation time compared to single-block models on these simulations.

The proposed approaches also generated promising results on real OMICs data (Petrovic et al., 2022). The development of stability-enhanced models accommodating data with a known block structure we proposed was triggered by the multi-OMICs application for the characterisation of the molecular signature of smoking. Their application to methylation and gene expression data gave further insights on the long-range correlations previously reported (Guida et al., 2015), and revealed a credible pivotal cross-OMICs role of the LRRN3 transcript (Huan et al., 2016). Annotation of the networks using biological information from the Reactome database identifies modules mostly composed of nodes belonging to the same pathways, suggesting that statistical correlations can reflect functional role in shared biological pathways.

The stability selection approach and calibration procedure introduced here could also be used in combination with other variable selection algorithms, including penalised unsupervised models that cannot rely on the minimisation of an error term in cross-validation (Zou et al., 2006), or extensions modelling longitudinal (Charbonnier et al., 2010) or count data (Chiquet et al., 2018). The method and its implementation in the R package sharp comes with some level of flexibility and user-controlled choices. Depending on the application and its requirements, the models can be tailored to generate more or less conservative results using (a) the threshold in PFER controlling the sparsity of the selected sets and (b) considering features with intermediate selection proportions (between 1−π and *π*). The calculation of our stability score can alternatively be based on two categories including (a) stably selected features with $H_{\lambda }(j)\geq K\pi$, and (b) non-stably selected features with $H_{\lambda }(j)<K\pi$. As this definition would ignore stably excluded features, which also contribute to the overall model stability, it may hamper selection performances.

Nevertheless, the results of stability selection models should always be interpreted with care. Our simulation studies indicate that, even when the assumptions of the model are verified (including the multivariate Normal distribution), the estimations of the graphical models are not perfect. In particular, some of the edges selected may correspond to marginal relationships (and not true conditional links). On the other hand, the absence of an edge does not necessarily indicate that there is no conditional association between the two nodes (especially for cross-group edges, for which the signal is diluted). Reassuringly, the overall topology of the graph seems relevant, as observed when applied on data with a scale-free graphical structure.

Stability selection approaches are based on the assumption that important features are stable, i.e. frequently selected over multiple subsamples of the study population. In the presence of correlated features that could be used interchangeably in the model for no loss of prediction performance (Breiman, 2001), selection proportions are naturally reduced due to the competition across correlated features. As suggested by our simulation study with correlated predictors, the use of our stability score for calibration may help in detecting all relevant features even if they are correlated by identifying the most stable model, which would include all surrogates.

As with all penalised approaches, the stability selection models we propose rely on a sparsity assumption. In regression, this assumption implies that some of the predictors do not contribute to the prediction of the outcome or provide information that is redundant with that from other predictors. As the stability score $S_{\lambda ,\pi }$ we propose is equal to zero the stability selection model is empty (no stably selected features) or saturated (all features are stably selected), our calibration procedure is only informative for models where the number of stably selected features is between 1 and (N−1). The validity of this sparsity assumption could be investigated post-hoc using unpenalised models sequentially adding the selected features in decreasing order of selection proportion.

Our calibration procedure is solely based on stability and does not rely on prediction performance, even for regression models. As such, there is no guarantee that stably selected variables are the most predictive ones. To assess the predictive performances of the stability selection model and to evaluated the per-feature contribution to these performances, we recommend to complement stability selection by post-hoc evaluation of prediction performances where features are incrementally added as predictors in the model in decreasing order of selection proportion, as illustrated in this article.

The calculation of the stability score relies on the assumption that the feature selection counts are independent. The link between correlation across features and correlation of their selection counts is not obvious and would warrant further investigation. However, selection and prediction performances of our calibrated stability selection LASSO models do not seem to be affected by the presence of correlated predictors.

While stability selection LASSO has been successfully applied on high-dimensional data with almost 450,000 predictors (Petrovic et al., 2022), the stability selection graphical LASSO has limited scalability. The complexity of graphical models is rapidly increasing with the number of nodes, and despite recent faster implementations of the graphical models (Sustik & Calderhead, 2012), computation times remain high with more than a few hundreds of nodes. Beyond their computational burden, large graphical models can become very dense and more efficient ways of visualising and summarising the results will be needed. Alternatively, as structures of redundant interconnected nodes (cliques) can be observed, summarising these in super-nodes could be valuable. This could be achieved using clustering or dimensionality reduction approaches, or by incorporating a priori biological knowledge in the model.

## Supplementary Material

qlad058_Supplementary_Data

## Data Availability

Data sharing is not applicable to this article as no new data were created or analysed in this study. All codes and simulated datasets are available on https://github.com/barbarabodinier/stability˙selection. The R packages sharp and fake are available on the Comprehensive R Archive Network (CRAN).
