# Supplementary material for: Automated calibration for stability selection in penalised regression and graphical models
Source: J R Stat Soc Ser C Appl Stat. 2023 Jul 13;72(5):1375–93. doi: 10.1093/jrsssc/qlad058 (PMC10746547; doi:10.1093/jrsssc/qlad058)
Supplement: qlad058_Supplementary_Data [file qlad058_supplementary_data.pdf]

Supplementary Material: Automated calibration for stability selection in penalised regression and graphical models

Barbara Bodinier

MRC Centre for Environment and Health, Department of Epidemiology and Biostatistics, School of Public Health, Imperial College London, London, United Kingdom.

E-mail: b.bodinier@imperial.ac.uk

Sarah Filippi

Department of Mathematics, Imperial College London, London, UK.

Therese Haugdahl Nøst

Systemsepidemiology, Department of Community Medicine, UiT The Arctic university of Norway, Tromsø, Norway.

Julien Chiquet

Université Paris-Saclay, AgroParisTech INRAE, UMR MIA, Paris, France.

Marc Chadeau-Hyam

MRC Centre for Environment and Health, Department of Epidemiology and Biostatistics, School of Public Health, Imperial College London, London, United Kingdom.

1. Supplementary Methods: existing calibration strategies

In both LASSO-regularised regression and graphical modelling, the calibration of the hyper-parameter λ is critical as it regulates the size of the set of selected features. State-of-the-art approaches for the choice of λ are based on M-fold cross-validation minimising some error metric (e.g. Mean Squared Error in Prediction). For graphical models, information theory metrics are commonly used, including the Akaike, Bayesian, and Extended Bayesian Information Criterion (Akaike, 1998; Schwarz, 1978; Foygel and Drton, 2010; Chiquet et al., 2009):

$$\mathrm{AIC}_\lambda = -2\ell(\hat{\Omega}_\lambda) + 2|E_\lambda|$$

$$\mathrm{BIC}_\lambda = -2\ell(\hat{\Omega}_\lambda) + \log(n)|E_\lambda|$$

$$\mathrm{EBIC}_\lambda = -2\ell(\hat{\Omega}_\lambda) + \log(n)\left(|E_\lambda| + 4\gamma\log(p)\right)$$

where $\ell(\hat{\Omega}_\lambda) = \frac{n}{2}\log\det(\hat{\Omega}_\lambda) - \mathrm{tr}(\hat{\Omega}_\lambda S)$ is the penalised likelihood, $|E_\lambda|$ is the degrees of freedom (i.e. number of edges in the graph), and γ is a hyper-parameter specific to the EBIC.

References

Akaike, H. (1998). *Information Theory and an Extension of the Maximum Likelihood Principle*, pp. 199–213. New York, NY: Springer New York.

Chiquet, J., A. Smith, G. Grasseau, C. Matias, and C. Ambroise (2009, February). SIMoNe: Statistical Inference for MOdular NEtworks. *Bioinformatics -Oxford- 25*(3), 417–8.

Foygel, R. and M. Drton (2010). Extended bayesian information criteria for gaussian graphical models. In J. D. Lafferty, C. K. I. Williams, J. Shawe-Taylor, R. S. Zemel, and A. Culotta (Eds.), *Advances in Neural Information Processing Systems 23*, pp. 604–612. Curran Associates, Inc.

Schwarz, G. (1978, 03). Estimating the dimension of a model. *Ann. Statist. 6*(2), 461–464.

2. Supplementary Figures and Tables

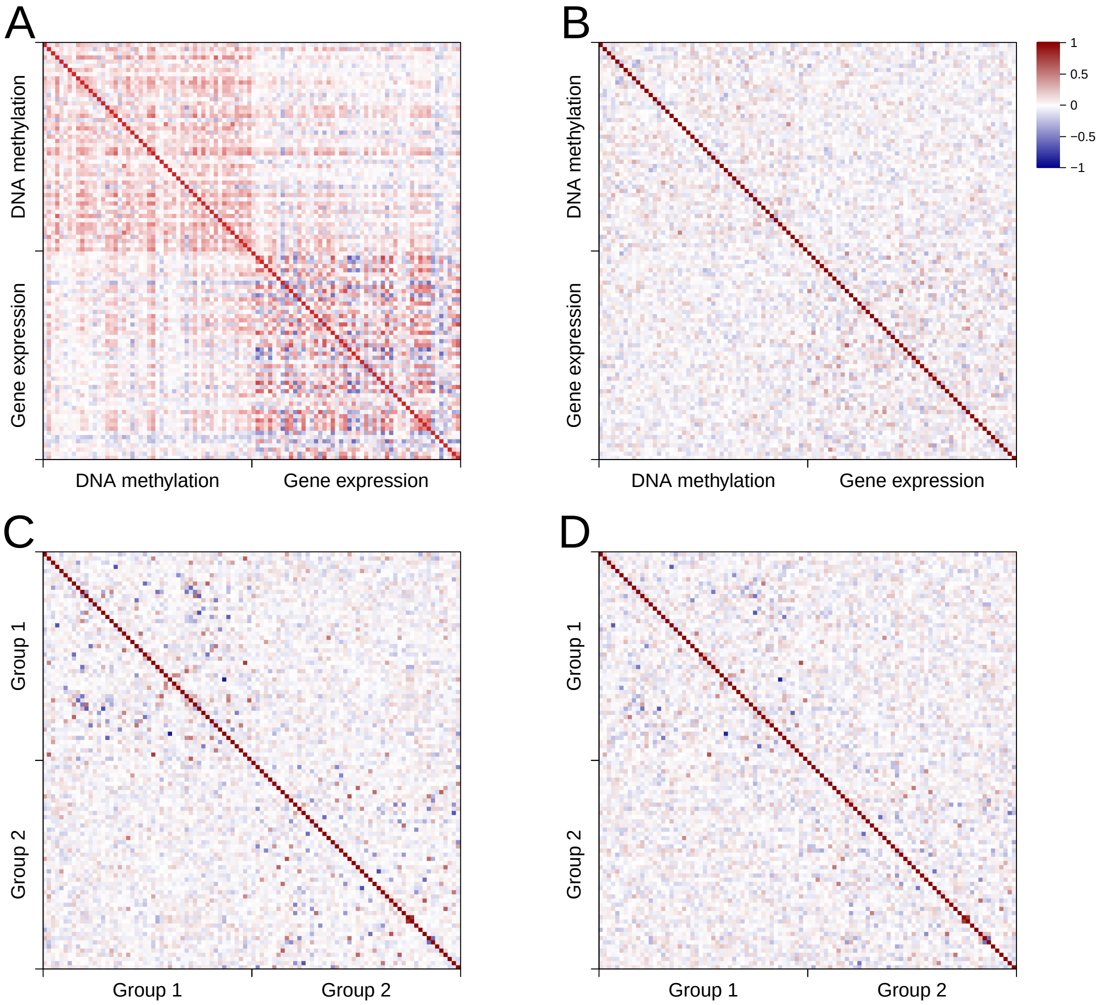

Supplementary Figure S1: Simulation of data with a block correlation structure. The heatmaps show Pearson's correlation (A) and partial correlation (B) matrices estimated on real data from 50 randomly chosen DNA methylation and gene expression markers. The bottom panel shows Pearson's correlation (C) and partial correlation (D) matrices estimated on simulated data with $n = 200$ observations and a block structure (50 variables in each group). The simulated conditional independence structure between the $p = 100$ variables is that of a random graph ($\nu = 0.04$, $v_b = 0.2$). All partial correlations are estimated without penalisation.

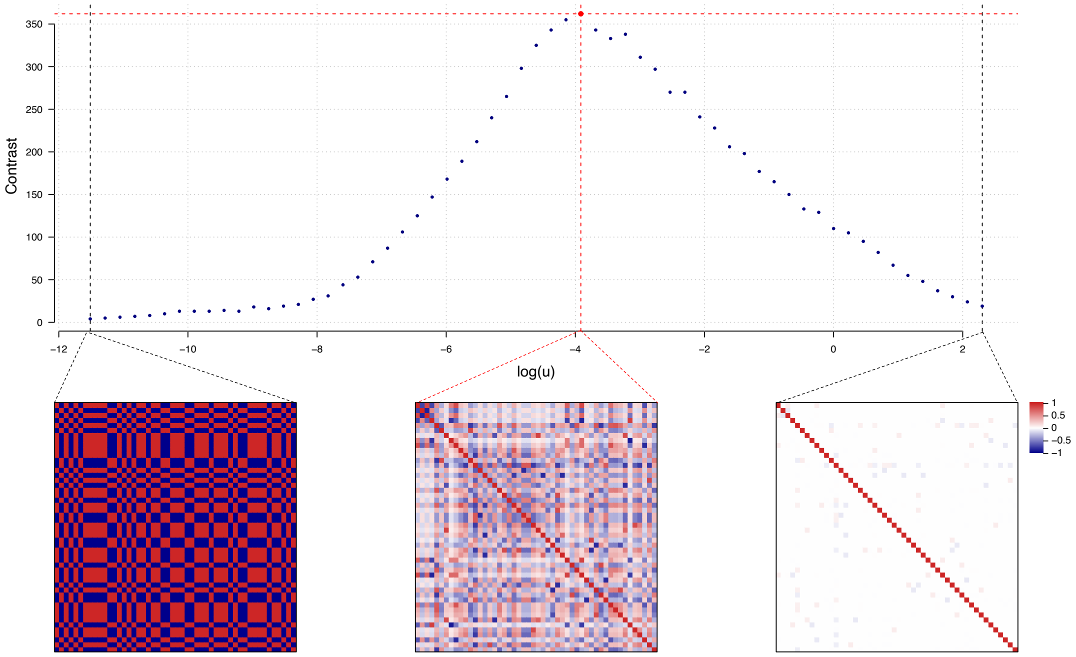

Supplementary Figure S2: Choice of the value of parameter u for simulation of the precision matrix. The contrast of the simulated correlation matrix for a scale-free graphical model with $p = 50$ nodes and $n = 100$ observations is represented as a function of the parameter u on the log-scale. The chosen value for u is the one maximising the contrast (indicated by a red dashed line). The heatmaps of correlation matrices with extreme and calibrated values of the parameter u are showed.

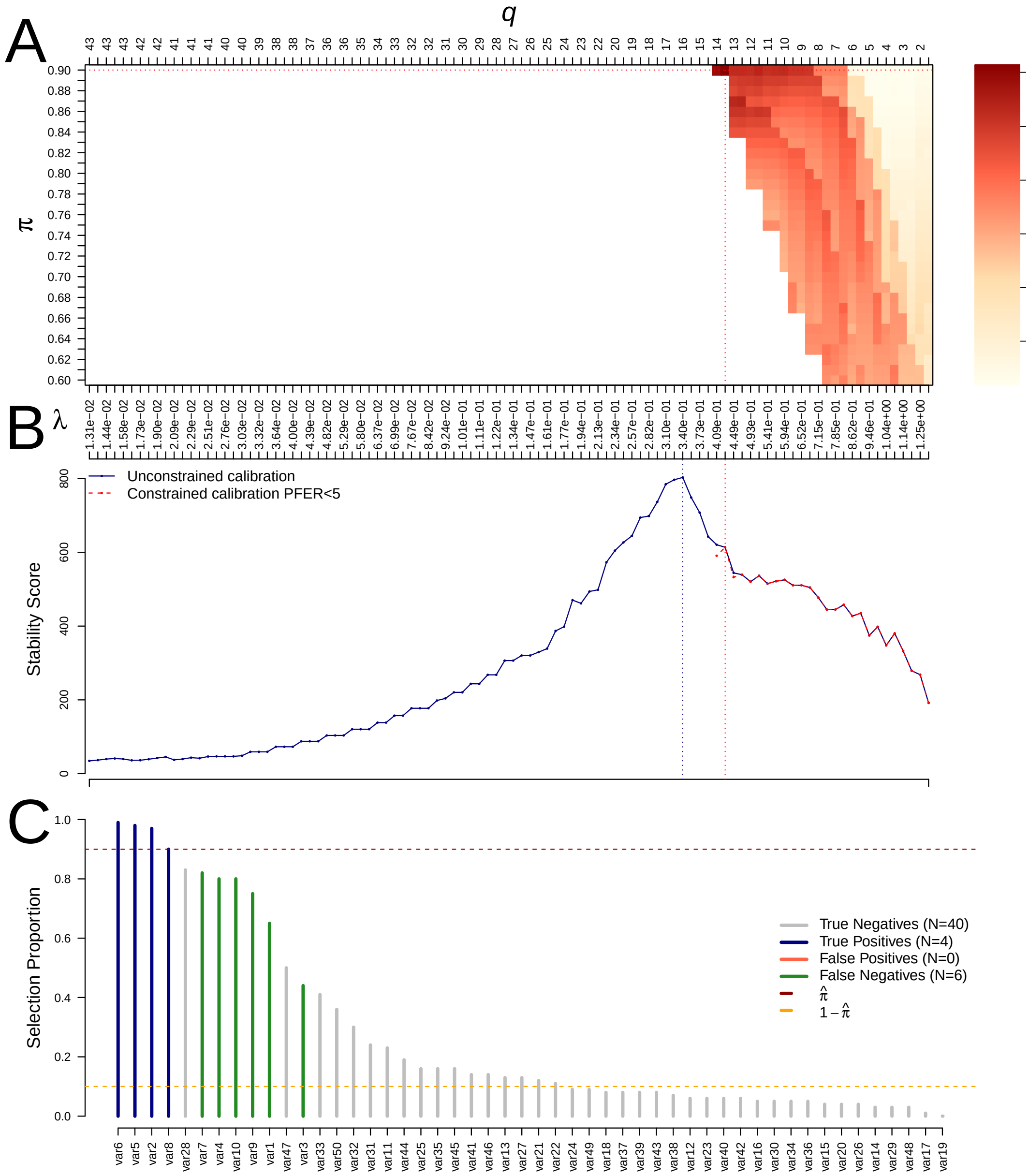

Supplementary Figure S3: Visualisation of the PFER constraint in calibration of stability selection models. The calibration heatmap shows the stability score (colour-coded) as a function of λ (or the corresponding average number of selected variables q) and π (A). The white area (left) represents models for which the PFER computed using the Meinshausen and Bühlmann approach would exceed the threshold ($\text{PFER}_{MB} > 5$). The highest stability score obtained for a given penalty parameter λ is represented for the unconstrained (blue) and constrained (red dotted line) approaches (B). Ordered selection proportions obtained from constrained calibration are reported (C). Stability selection is applied on simulated data with $n = 100$ observations for $p = 50$ variables, of which 10 contribute to the definition of the outcome with effect sizes in $\{[-1, -0.5] \cup [0.5, 1]\}$ and an expected proportion of explained variance of 70%.

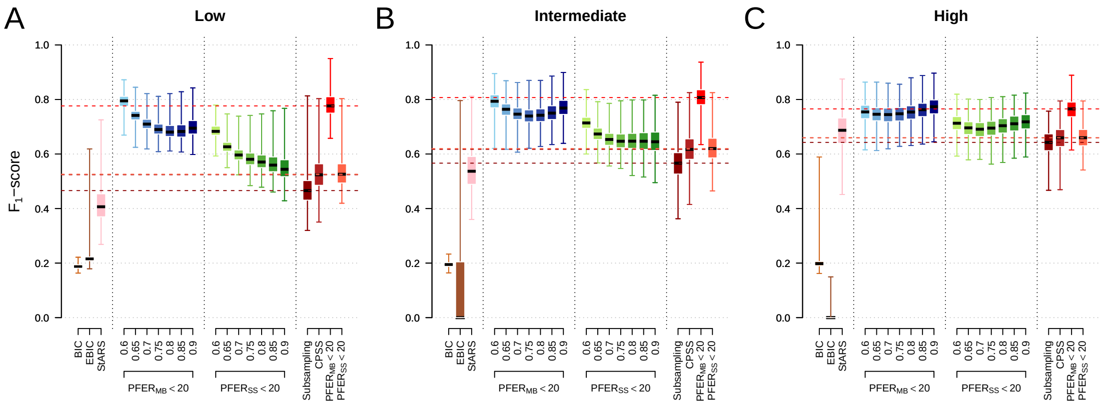

Supplementary Figure S4: Selection performances of state-of-the-art approaches and proposed calibrated stability selection graphical LASSO models applied on simulated data with scale-free underlying graph structure. We show the median, quartiles, minimum and maximum F_1-score of graphical LASSO models calibrated using the BIC, EBIC, StARS, and stability selection graphical LASSO models calibrated via error control (MB in blue, SS in green) or using the proposed stability score (red). Models are applied on 1,000 simulated datasets with $p = 100$ variables following a multivariate Normal distribution corresponding to a random graph structure ($\nu = 0.02$). Performances are estimated in low ($n = 2p = 200$), intermediate ($n = p = 100$), and high ($n = p/2 = 50$) dimensions.

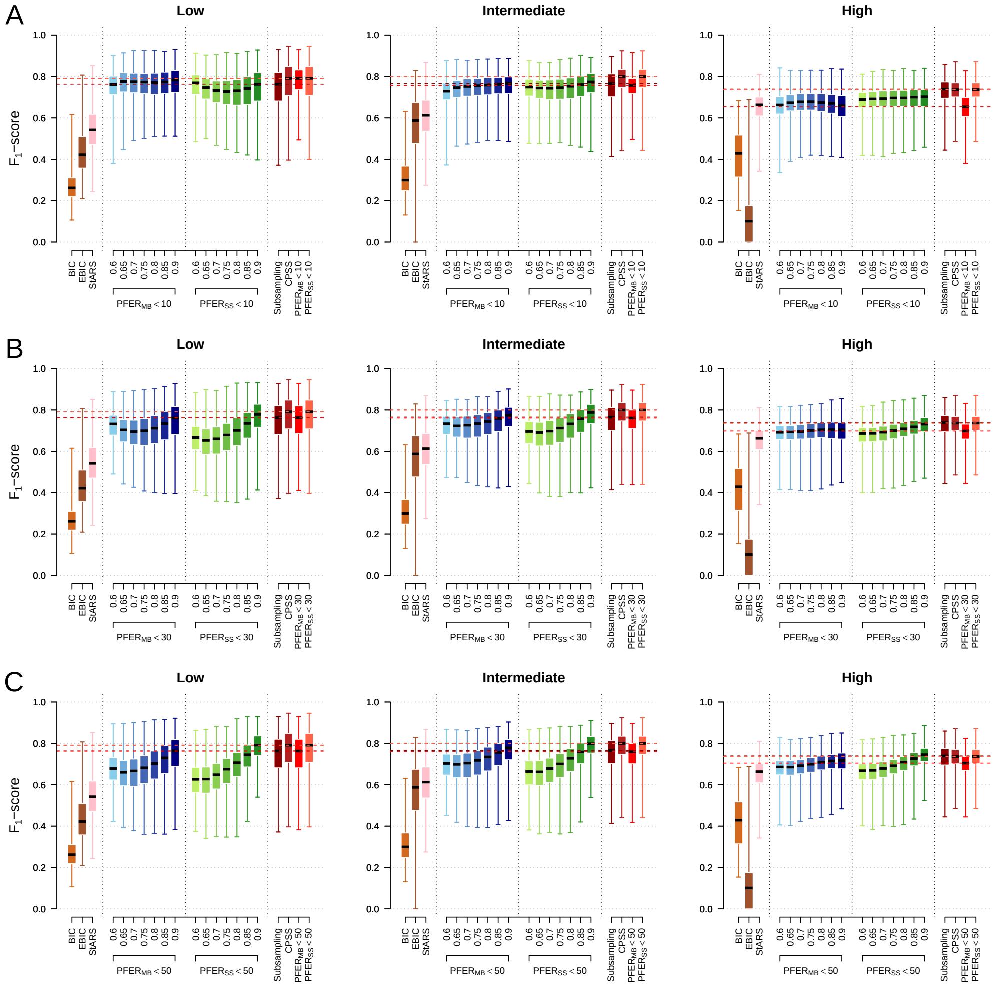

Supplementary Figure S5: Selection performances of state-of-the-art approaches and proposed calibrated stability selection graphical LASSO models with different thresholds in PFER. We show the median, quartiles, minimum and maximum F_1-score of graphical LASSO models calibrated using the BIC, EBIC, StARS, and stability selection graphical LASSO models calibrated via error control (MB in blue, SS in green) or using the proposed stability score (red). The threshold in PFER for stability selection models was set to 10 (A), 30 (B) or 50 (C). Models are applied on 1,000 simulated datasets with $p = 100$ variables following a multivariate Normal distribution corresponding to a random graph structure ($\nu = 0.02$). Performances are estimated in low ($n = 2p = 200$), intermediate ($n = p = 100$), and high ($n = p/2 = 50$) dimensions.

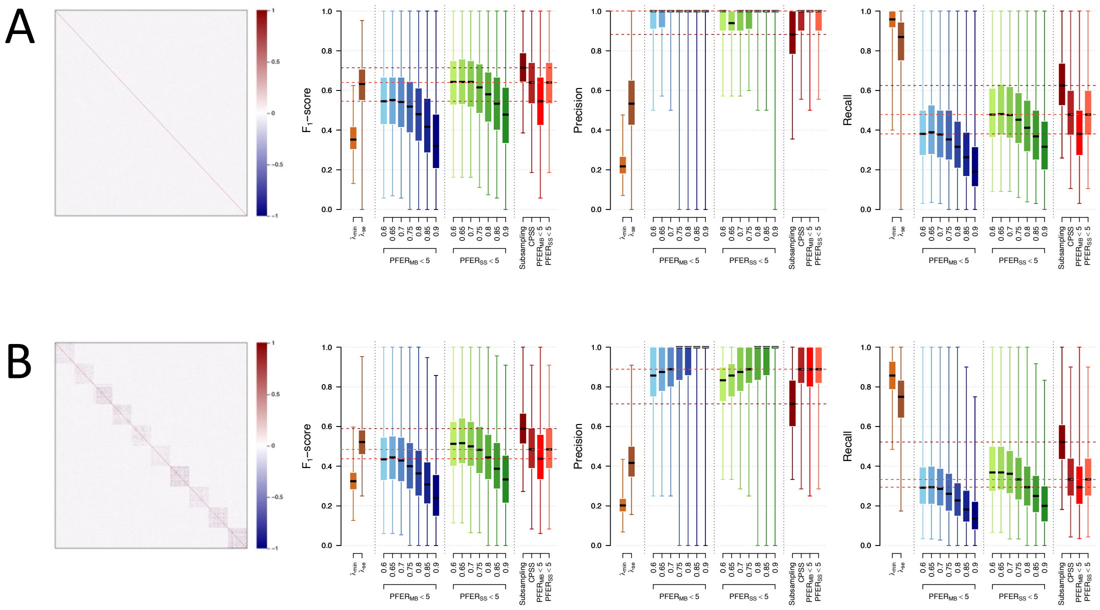

Supplementary Figure S6: Selection performances of state-of-the-art approaches and proposed calibrated stability selection LASSO models applied on simulated data. Models are applied on 1,000 simulated datasets with $n = 500$ observations and $p = 1,000$ predictor variables, of which an expected proportion $\nu_Y = 0.02$ contributes to the definition of the outcome with effect sizes in $\{-1, 1\}$ and an expected proportion of explained variance of 40%. We simulate independent predictors, conditionally on the outcome (A) or blocks of correlated predictors where the conditional independence structure within blocks is that of a random network of density $\nu = 0.02$ (B). For both settings, we represent a heatmap of Pearson's correlations between predictors in a typical simulation. We show the median, quartiles, minimum and maximum F_1-score, precision and recall of LASSO models calibrated by 10-fold cross validation minimising the Mean Squared Error of Prediction (λ_{min}) or one standard error away from the minimum (λ_{se}), and stability selection LASSO models calibrated via error control (MB in blue, SS in green) or using the proposed stability score (red).

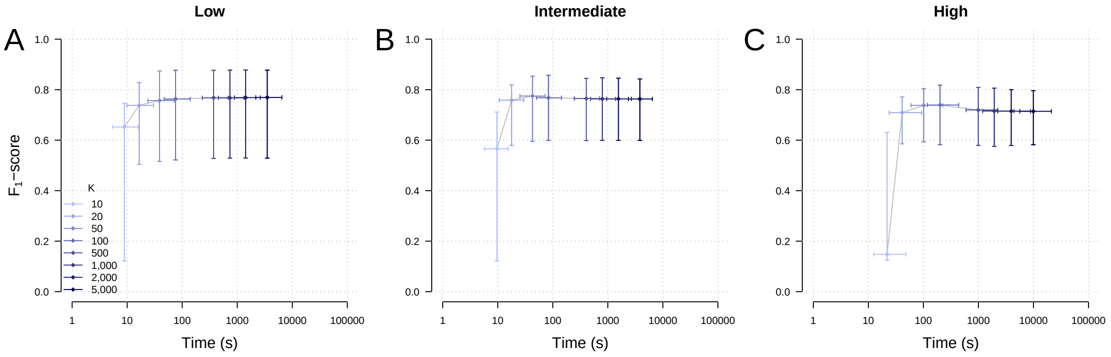

Supplementary Figure S7: Effect of the number of subsampling iterations K on the selection performance and computation time. The median, 5th and 95th quantile of the F_1-score and computation time are reported for graphical LASSO stability selection models calibrated using the unconstrained approach and with different numbers of iterations K (10, 20, 50, 100, 500, 1,000, 2,000, and 5,000). The models are applied on simulated data ($p = 100$) with underlying random graph structure ($\nu = 0.02$). The computation time in seconds is reported on the log-scale (X-axis). Performances are evaluated in low ($n = 2p = 200$), intermediate ($n = p = 100$), and high ($n = p/2 = 50$) dimensions.

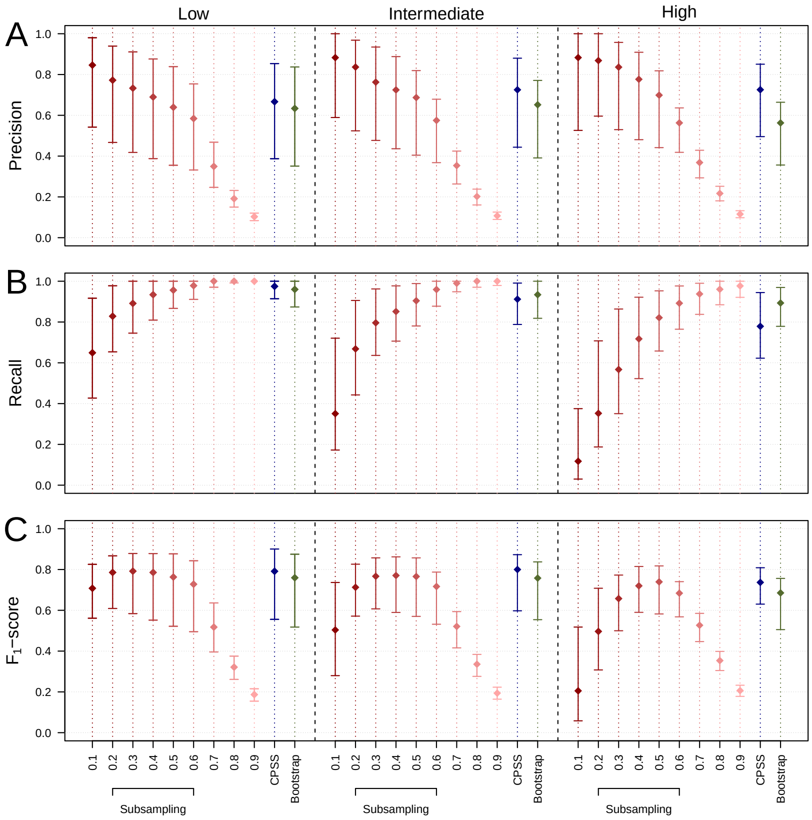

Supplementary Figure S8: Effect of the choice of resampling technique on the selection performance. The median, 5th and 95th quantile of the F_1-score are reported for stability selection models calibrated using the unconstrained approach with different resampling approaches: subsampling with different subsample sizes τ between 0.1 and 0.9 (red), simultaneous selection in complementary pairs (CPSS, in dark blue) and bootstraping (resampling with replacement, dark green). Performances are evaluated in low ($n = 2p = 200$), intermediate ($n = p = 100$), and high ($n = p/2 = 50$) dimensions

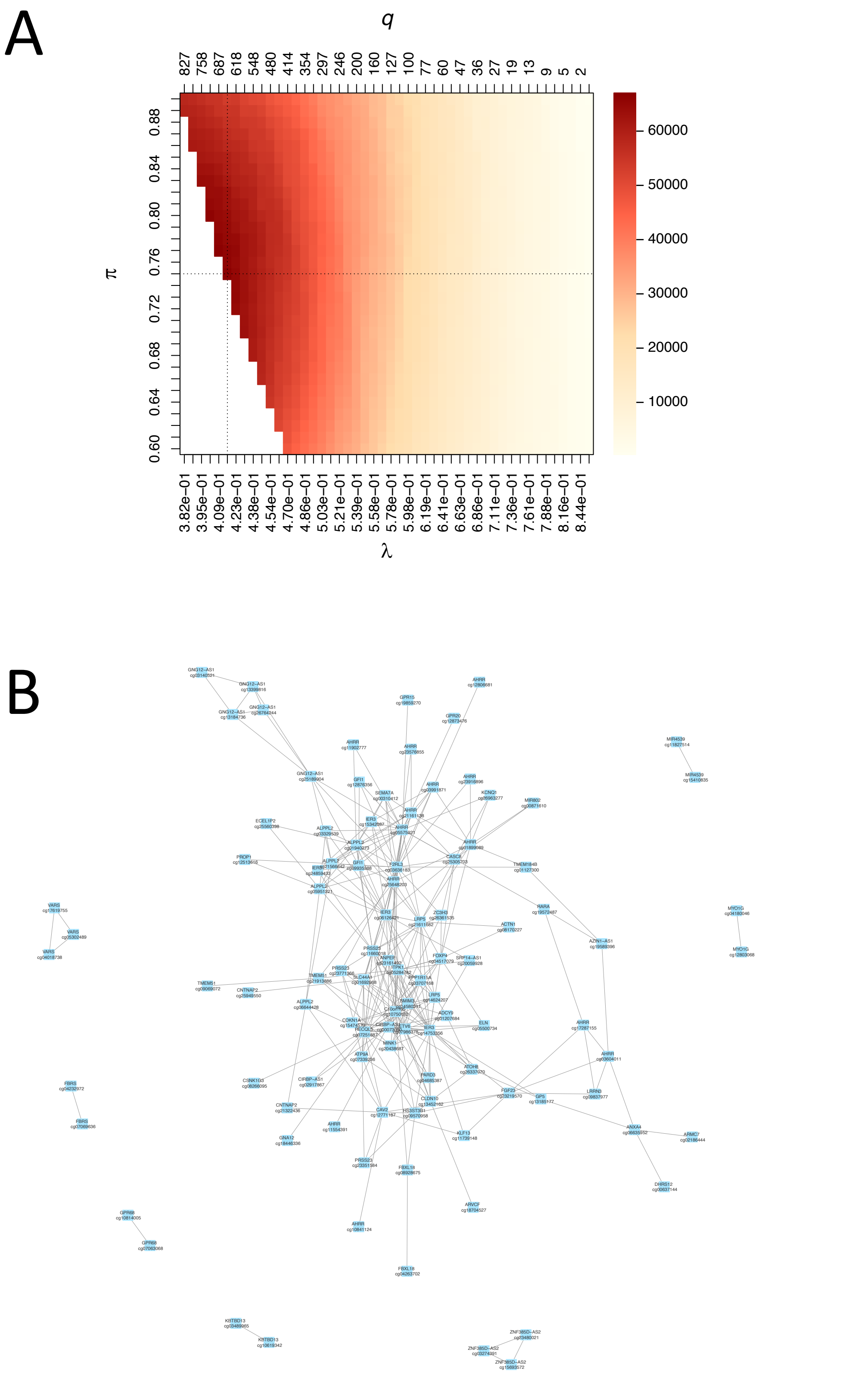

Supplementary Figure S9: Single-block graphical model of DNA methylation markers of exposure to tobacco smoking. Calibration is done by maximising the stability score while ensuring that $\mathrm{PFER}_{MB} < 70$ (A). CpG sites with at least one edge are represented in the graph (B).

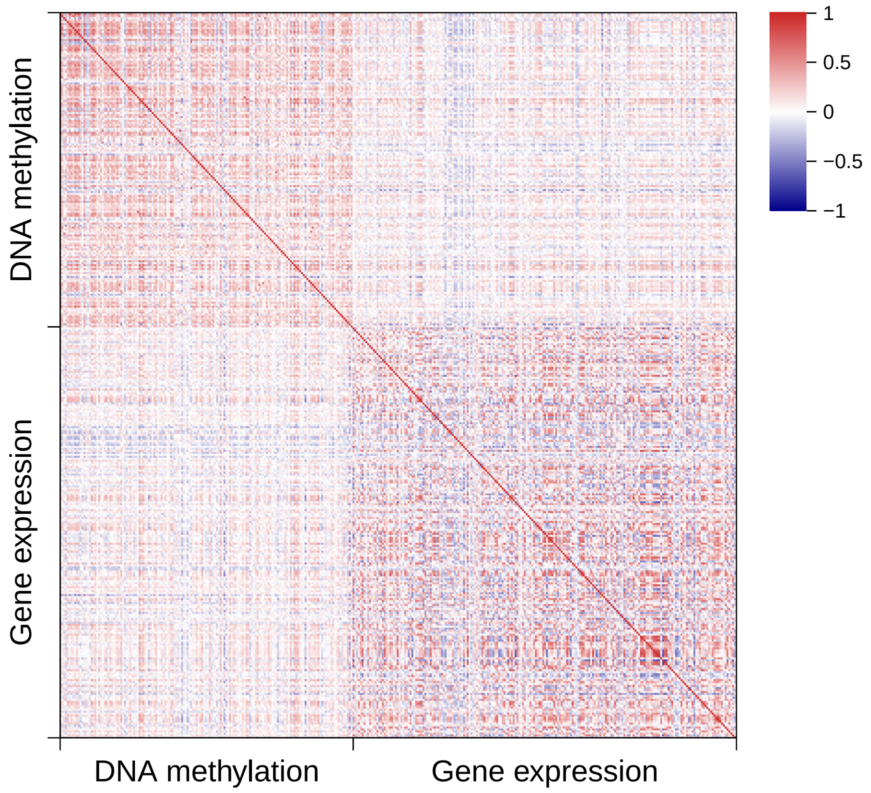

Supplementary Figure S10: Heatmap of Pearson's correlations estimated from measured levels of the 159 DNA methylation markers and 208 gene expression markers.

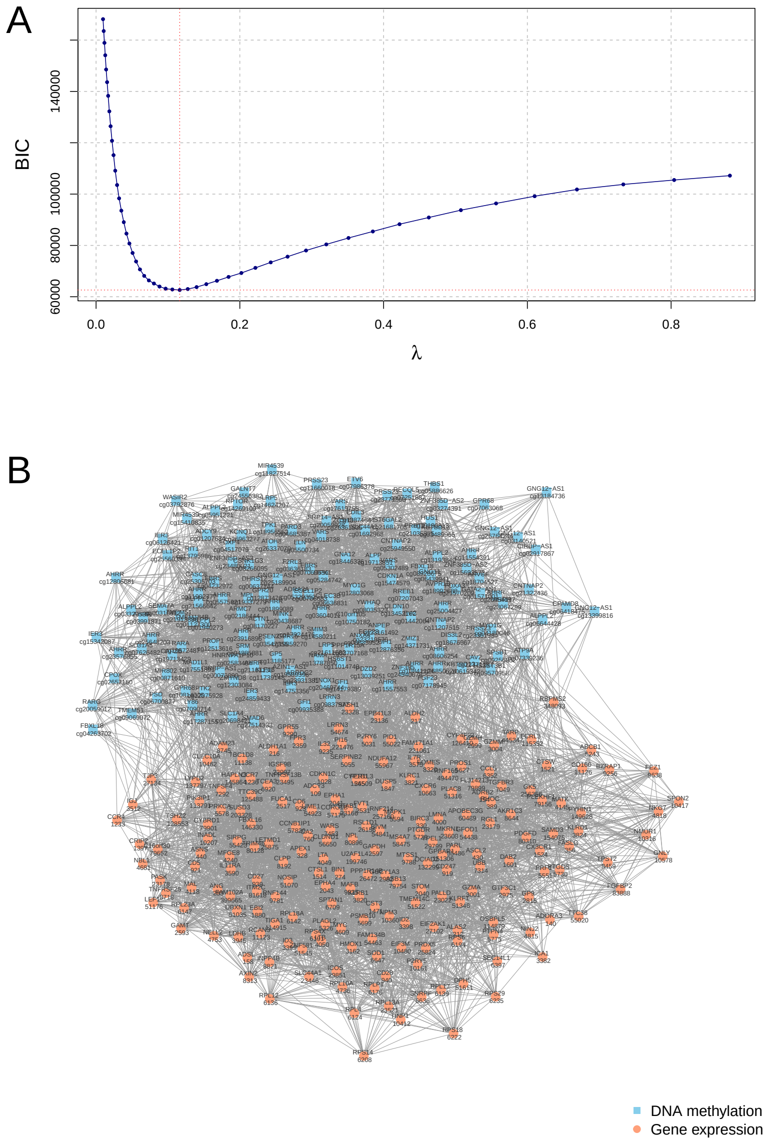

Supplementary Figure S11: Graphical LASSO model of smoking-related methylation (blue square) and gene expression (red circle) markers calibrated using the Bayesian Information Criterion (BIC). The BIC is represented as a function of the penalty parameter λ (A). The graphical model generating the smallest BIC is showed (B).

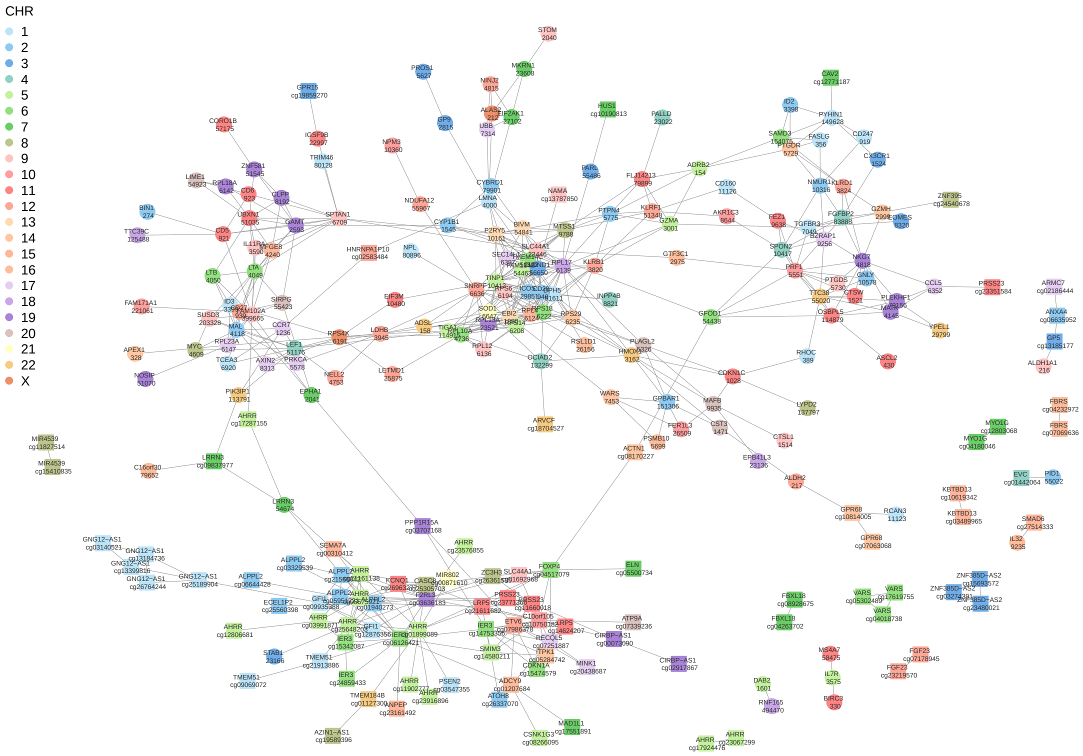

Supplementary Figure S12: Multi-OMICs graphical model integrating DNA methylation (square) and gene expression (circle) markers of tobacco smoking with nodes coloured by chromosome.

		π	TP	FP	FN	Precision	Recall	F_1-score	Time (s)
	AIC		99 [13]	1959 [448]	0 [0]	0.049 [0.016]	1.000 [0.000]	0.093 [0.030]	1 [0]
	BIC		99 [13]	564 [205]	0 [0]	0.150 [0.061]	1.000 [0.000]	0.260 [0.091]	1 [0]
	EBIC		98 [13]	264 [148]	0 [0]	0.271 [0.125]	1.000 [0.000]	0.426 [0.152]	1 [0]
	StARS		98 [12]	162 [84]	1 [1]	0.373 [0.143]	0.991 [0.012]	0.543 [0.148]	78 [39]
		0.6	89 [12]	44 [19]	10 [7]	0.668 [0.124]	0.897 [0.072]	0.768 [0.093]	83 [40]
		0.65	92 [12]	58 [25]	6 [5]	0.616 [0.123]	0.936 [0.055]	0.743 [0.096]	84 [38]
		0.7	94 [11]	66 [30]	5 [5]	0.590 [0.136]	0.953 [0.048]	0.729 [0.105]	86 [38]
	MB	0.75	95 [11]	68 [36]	4 [4]	0.582 [0.150]	0.962 [0.041]	0.725 [0.116]	87 [38]
		0.8	95 [12]	66 [39]	3 [4]	0.590 [0.161]	0.968 [0.038]	0.731 [0.124]	87 [38]
		0.85	95 [10]	62 [40]	3 [5]	0.605 [0.171]	0.969 [0.044]	0.746 [0.127]	88 [38]
L		0.9	95 [10]	56 [41]	3 [5]	0.633 [0.187]	0.968 [0.044]	0.763 [0.131]	88 [37]
		0.6	94 [12]	74 [28]	4 [5]	0.562 [0.123]	0.958 [0.045]	0.708 [0.102]	87 [36]
		0.65	96 [12]	88 [39]	2 [3]	0.522 [0.134]	0.978 [0.031]	0.680 [0.114]	86 [35]
		0.7	97 [11]	90 [47]	2 [3]	0.519 [0.150]	0.981 [0.032]	0.678 [0.129]	86 [34]
	SS	0.75	97 [12]	87 [50]	2 [3]	0.531 [0.160]	0.983 [0.029]	0.688 [0.135]	85 [34]
		0.8	97 [12]	80 [50]	1 [3]	0.551 [0.166]	0.989 [0.029]	0.706 [0.136]	84 [32]
		0.85	97 [12]	70 [46]	1 [2]	0.582 [0.170]	0.990 [0.024]	0.733 [0.136]	84 [32]
		0.9	97 [12]	58 [38]	1 [2]	0.631 [0.173]	0.990 [0.021]	0.770 [0.124]	83 [32]
	Subsampling	0.9	94 [10]	54 [46]	4 [5]	0.640 [0.203]	0.957 [0.052]	0.764 [0.137]	81 [32]
	CPSS	0.9	96 [11]	48 [40]	3 [4]	0.669 [0.199]	0.973 [0.039]	0.793 [0.138]	83 [33]
	MB	0.9	93 [10]	52 [39]	5 [5]	0.645 [0.185]	0.949 [0.055]	0.769 [0.126]	81 [32]
	SS	0.9	96 [11]	48 [40]	3 [4]	0.669 [0.199]	0.973 [0.039]	0.793 [0.138]	82 [31]
	AIC		98 [13]	1760 [813]	0 [0]	0.053 [0.028]	1.000 [0.000]	0.101 [0.050]	1 [0]
	BIC		98 [12]	460 [223]	0 [1]	0.176 [0.084]	1.000 [0.010]	0.299 [0.119]	1 [0]
	EBIC		94 [10]	129 [107]	3 [5]	0.426 [0.213]	0.972 [0.050]	0.589 [0.198]	1 [0]
	StARS		94 [10]	115 [75]	3 [5]	0.451 [0.171]	0.966 [0.053]	0.614 [0.151]	82 [42]
		0.6	83 [8]	39 [20]	16 [10]	0.679 [0.128]	0.837 [0.087]	0.748 [0.083]	88 [41]
		0.65	86 [9]	47 [26]	12 [9]	0.648 [0.140]	0.876 [0.079]	0.743 [0.088]	91 [39]
		0.7	87 [8]	50 [30]	11 [9]	0.636 [0.150]	0.893 [0.080]	0.741 [0.092]	95 [40]
	MB	0.75	88 [9]	50 [33]	10 [9]	0.640 [0.163]	0.898 [0.084]	0.743 [0.096]	96 [39]
		0.8	88 [9]	47 [34]	10 [9]	0.655 [0.170]	0.901 [0.086]	0.752 [0.097]	95 [38]
		0.85	87 [8]	43 [34]	10 [10]	0.672 [0.176]	0.897 [0.090]	0.761 [0.094]	95 [37]
I		0.9	86 [8]	37 [32]	11 [11]	0.702 [0.176]	0.887 [0.102]	0.773 [0.086]	95 [38]
		0.6	89 [10]	60 [31]	9 [7]	0.600 [0.139]	0.909 [0.072]	0.721 [0.095]	95 [37]
		0.65	91 [10]	66 [39]	7 [7]	0.580 [0.151]	0.931 [0.065]	0.711 [0.106]	95 [35]
		0.7	92 [10]	67 [42]	6 [7]	0.583 [0.161]	0.939 [0.063]	0.716 [0.113]	94 [37]
	SS	0.75	92 [10]	63 [43]	6 [7]	0.595 [0.167]	0.941 [0.062]	0.725 [0.114]	94 [38]
		0.8	92 [10]	58 [42]	6 [7]	0.615 [0.174]	0.942 [0.065]	0.738 [0.112]	94 [37]
		0.85	92 [9]	52 [40]	6 [7]	0.642 [0.180]	0.941 [0.069]	0.757 [0.111]	94 [39]
		0.9	91 [9]	44 [35]	6 [7]	0.681 [0.176]	0.941 [0.071]	0.782 [0.098]	94 [37]
	Subsampling	0.9	88 [10]	40 [33]	9 [9]	0.687 [0.182]	0.905 [0.086]	0.765 [0.108]	90 [39]
	CPSS	0.9	89 [10]	34 [29]	8 [10]	0.725 [0.171]	0.912 [0.088]	0.800 [0.088]	91 [38]
	MB	0.89	84 [10]	36 [30]	13 [12]	0.704 [0.166]	0.864 [0.107]	0.763 [0.086]	89 [36]
	SS	0.9	89 [10]	34 [29]	8 [10]	0.725 [0.171]	0.912 [0.088]	0.800 [0.088]	89 [35]
	AIC		97 [12]	3115 [125]	1 [2]	0.030 [0.004]	0.989 [0.022]	0.059 [0.007]	2 [1]
	BIC		93 [9]	242 [240]	4 [8]	0.279 [0.173]	0.961 [0.075]	0.431 [0.203]	2 [1]
	EBIC		5 [10]	0 [1]	92 [12]	0.854 [1.000]	0.054 [0.097]	0.101 [0.175]	2 [1]
	StARS		80 [9]	60 [52]	17 [17]	0.574 [0.185]	0.830 [0.154]	0.664 [0.092]	217 [101]
		0.6	69 [8]	31 [22]	29 [17]	0.694 [0.143]	0.708 [0.140]	0.689 [0.075]	214 [100]
		0.65	72 [9]	34 [25]	26 [17]	0.681 [0.149]	0.733 [0.150]	0.691 [0.072]	211 [105]
		0.7	72 [9]	34 [26]	25 [18]	0.683 [0.150]	0.742 [0.156]	0.695 [0.072]	215 [102]
	MB	0.75	72 [10]	32 [27]	26 [19]	0.689 [0.157]	0.737 [0.164]	0.696 [0.075]	214 [103]
		0.8	71 [10]	30 [26]	27 [20]	0.705 [0.161]	0.728 [0.176]	0.697 [0.075]	215 [98]
		0.85	69 [11]	26 [25]	29 [21]	0.726 [0.166]	0.704 [0.187]	0.696 [0.080]	213 [102]
H		0.9	66 [13]	22 [22]	32 [23]	0.753 [0.158]	0.670 [0.196]	0.692 [0.085]	210 [102]
		0.6	76 [8]	43 [28]	21 [16]	0.639 [0.144]	0.780 [0.137]	0.692 [0.072]	206 [100]
		0.65	78 [8]	47 [33]	19 [15]	0.629 [0.153]	0.803 [0.137]	0.692 [0.071]	200 [99]
		0.7	78 [8]	45 [33]	19 [17]	0.636 [0.158]	0.808 [0.145]	0.696 [0.070]	196 [101]
	SS	0.75	78 [9]	43 [33]	19 [16]	0.648 [0.163]	0.802 [0.150]	0.699 [0.069]	194 [99]
		0.8	77 [10]	39 [31]	20 [18]	0.667 [0.157]	0.792 [0.160]	0.706 [0.072]	190 [97]
		0.85	76 [10]	34 [29]	21 [19]	0.688 [0.156]	0.782 [0.171]	0.713 [0.071]	190 [99]
		0.9	75 [11]	29 [28]	23 [20]	0.721 [0.156]	0.771 [0.178]	0.720 [0.077]	189 [98]
	Subsampling	0.9	80 [10]	35 [23]	17 [13]	0.699 [0.141]	0.822 [0.119]	0.740 [0.079]	189 [95]
	CPSS	0.86	76 [9]	29 [18]	22 [15]	0.726 [0.112]	0.779 [0.135]	0.737 [0.067]	185 [89]
	MB	0.82	66 [11]	24 [21]	32 [21]	0.733 [0.147]	0.674 [0.175]	0.689 [0.078]	185 [85]
	SS	0.86	76 [9]	29 [18]	22 [15]	0.726 [0.112]	0.779 [0.135]	0.737 [0.067]	182 [80]

Supplementary Table S1: Median and inter-quartile range of the selection performance metrics and computation times obtained with different graphical models. Models are applied on 1,000 simulated datasets with $p = 100$ variables following a multivariate Normal distribution corresponding to a random graph structure ($\nu = 0.02$) in low (L, $n = 2p = 200$), intermediate (I, $n = p = 100$), and high (H, $n = p/2 = 50$) dimensions.

LASSO		Graphical LASSO		
p		p	Cold start	Warm start
1,000	18 [5]	100	69 [33]	51 [22]
2,500	35 [9]	250	313 [104]	247 [73]
5,000	59 [20]	500	2,759 [1,163]	1,796 [658]
7,500	86 [35]	750	14,513 [5,983]	7,402 [3,472]
10,000	124 [63]	1,000	99,108 [29,563]	40,240 [10,787]

Supplementary Table S2: Median and inter-quartile range of the computation times (in seconds) of stability selection obtained with different numbers of variables p. Models are applied on 1,000 simulated datasets with $n = 500$ observations. For stability selection LASSO models, we use $p = 1,000$, $2,500$, $5,000$, $7,500$ or $10,000$ independent predictors, conditionally on the outcome. For stability selection graphical LASSO models, we use $p = 100$, 250, 500, 750 or $1,000$ variables following a multivariate Normal distribution corresponding to a random graph structure ($\nu = 0.02$). For graphical models, we report computation times with or without warm start, where models are iteratively fitted over a path from larger to smaller penalty values and the estimate from the previous iteration is a starting point for the gradient descent algorithm (argument "start" in the R package sharp). For LASSO models, we always use warm start as implemented in the R package glmnet.

			TP	FP	FN	Precision	Recall	F1-score	Time (s)
L	Single	Overall	84 [9]	79 [47]	13 [12]	0.521 [0.143]	0.863 [0.104]	0.643 [0.089]	96 [37]
		Within 1	24 [6]	24 [15]	0 [0]	0.500 [0.146]	1.000 [0.000]	0.663 [0.130]	
		Between	36 [11]	30 [28]	13 [11]	0.545 [0.191]	0.735 [0.204]	0.604 [0.105]	
		Within 2	24 [6]	24 [15]	0 [0]	0.500 [0.149]	1.000 [0.000]	0.667 [0.127]	
	Multi	Overall	93 [10]	73 [34]	6 [6]	0.561 [0.125]	0.941 [0.056]	0.703 [0.087]	269 [94]
		Within 1	24 [6]	16 [10]	0 [0]	0.585 [0.132]	1.000 [0.000]	0.737 [0.102]	
		Between	44 [8]	38 [25]	5 [5]	0.543 [0.169]	0.893 [0.107]	0.667 [0.110]	
		Within 2	24 [6]	16 [10]	0 [0]	0.587 [0.137]	1.000 [0.000]	0.737 [0.106]	
I	Single	Overall	77 [10]	62 [40]	22 [15]	0.557 [0.149]	0.782 [0.127]	0.643 [0.066]	107 [45]
		Within 1	24 [6]	19 [13]	0 [0]	0.550 [0.170]	1.000 [0.000]	0.704 [0.136]	
		Between	29 [11]	22 [22]	21 [14]	0.566 [0.184]	0.582 [0.244]	0.553 [0.107]	
		Within 2	24 [6]	19 [13]	0 [0]	0.554 [0.168]	1.000 [0.000]	0.708 [0.135]	
	Multi	Overall	86 [8]	62 [40]	12 [10]	0.583 [0.152]	0.873 [0.080]	0.697 [0.092]	310 [113]
		Within 1	24 [7]	11 [9]	0 [1]	0.674 [0.166]	1.000 [0.040]	0.800 [0.113]	
		Between	38 [8]	35 [31]	11 [8]	0.526 [0.202]	0.769 [0.146]	0.614 [0.141]	
		Within 2	24 [7]	11 [9]	0 [1]	0.676 [0.164]	1.000 [0.037]	0.800 [0.115]	
H	Single	Overall	71 [8]	47 [29]	28 [13]	0.597 [0.146]	0.716 [0.109]	0.641 [0.072]	242 [119]
		Within 1	24 [7]	14 [11]	0 [1]	0.606 [0.180]	1.000 [0.040]	0.746 [0.131]	
		Between	23 [9]	18 [14]	26 [12]	0.559 [0.154]	0.465 [0.198]	0.496 [0.110]	
		Within 2	23 [7]	14 [11]	0 [1]	0.617 [0.176]	1.000 [0.038]	0.750 [0.128]	
	Multi	Overall	77 [9]	71 [75]	21 [11]	0.519 [0.214]	0.787 [0.093]	0.627 [0.145]	534 [212]
		Within 1	23 [6]	9 [7]	1 [2]	0.719 [0.152]	0.966 [0.082]	0.812 [0.097]	
		Between	31 [8]	48 [77]	18 [8]	0.396 [0.306]	0.625 [0.146]	0.480 [0.225]	
		Within 2	23 [6]	9 [7]	1 [2]	0.710 [0.150]	0.967 [0.077]	0.812 [0.093]	

Supplementary Table S3: Median and inter-quartile range of the selection performance metrics and computation times obtained with single and multi-block stability selection applied on simulated data with a block structure. For each block, 50 different penalty parameter values are explored. Models are applied on 1,000 simulated datasets with $p = 100$ variables following a multivariate Normal distribution corresponding to a random graph ($\nu = 0.02$) and with known block structure (50 variables per group, using $v_b = 0.2$). Performances are evaluated in low (L, $n = 2p = 200$), intermediate (I, $n = p = 100$), and high (H, $n = p/2 = 50$) dimensions.

	λ_0		TP	FP	FN	Precision	Recall	F_1-score	Time (s)
S-B		Overall	85 [9]	78 [44]	14 [12]	0.523 [0.134]	0.859 [0.107]	0.646 [0.081]	70 [30]
		Within 1	25 [7]	24 [15]	0 [0]	0.500 [0.152]	1.000 [0.000]	0.667 [0.132]	
		Between	36 [11]	29 [27]	14 [11]	0.547 [0.187]	0.725 [0.209]	0.603 [0.099]	
		Within 2	24 [7]	23 [14]	0 [0]	0.504 [0.139]	1.000 [0.000]	0.667 [0.123]	
M-P		Overall	92 [11]	134 [55]	6 [6]	0.408 [0.109]	0.935 [0.060]	0.566 [0.098]	2059 [870]
		Within 1	24 [6]	11 [11]	0 [1]	0.679 [0.212]	1.000 [0.050]	0.800 [0.138]	
		Between	45 [8]	110 [51]	5 [4]	0.291 [0.094]	0.902 [0.092]	0.438 [0.099]	
		Within 2	23 [7]	11 [10]	0 [1]	0.677 [0.188]	1.000 [0.048]	0.794 [0.128]	
M-B	0	Overall	82 [8]	28 [48]	17 [10]	0.750 [0.266]	0.827 [0.094]	0.775 [0.136]	8493 [15464]
		Within 1	23 [6]	6 [5]	1 [3]	0.780 [0.148]	0.944 [0.115]	0.844 [0.089]	
		Between	36 [7]	14 [43]	13 [9]	0.720 [0.428]	0.737 [0.152]	0.697 [0.220]	
		Within 2	23 [7]	6 [5]	1 [3]	0.778 [0.152]	0.944 [0.115]	0.844 [0.091]	
	0.001	Overall	83 [8]	33 [65]	15 [11]	0.715 [0.323]	0.845 [0.093]	0.766 [0.184]	2966 [1577]
		Within 1	23 [6]	8 [8]	1 [2]	0.750 [0.178]	0.958 [0.094]	0.833 [0.104]	
		Between	37 [7]	16 [55]	12 [9]	0.705 [0.473]	0.755 [0.152]	0.689 [0.266]	
		Within 2	23 [6]	8 [7]	1 [2]	0.742 [0.169]	0.955 [0.096]	0.828 [0.101]	
	0.01	Overall	88 [9]	43 [49]	11 [9]	0.672 [0.230]	0.889 [0.075]	0.760 [0.135]	833 [288]
		Within 1	24 [6]	10 [8]	0 [1]	0.699 [0.175]	1.000 [0.045]	0.812 [0.120]	
		Between	40 [8]	20 [32]	10 [8]	0.671 [0.315]	0.807 [0.139]	0.714 [0.177]	
		Within 2	23 [7]	10 [8]	0 [1]	0.700 [0.165]	1.000 [0.048]	0.811 [0.113]	
	0.1	Overall	93 [11]	71 [33]	6 [7]	0.566 [0.123]	0.938 [0.058]	0.705 [0.081]	152 [51]
		Within 1	25 [6]	17 [10]	0 [0]	0.591 [0.143]	1.000 [0.000]	0.741 [0.109]	
		Between	44 [8]	37 [22]	6 [6]	0.550 [0.158]	0.884 [0.105]	0.671 [0.104]	
		Within 2	24 [7]	16 [10]	0 [0]	0.591 [0.133]	1.000 [0.000]	0.742 [0.101]	
	0.5	Overall	95 [11]	195 [90]	4 [5]	0.325 [0.107]	0.955 [0.048]	0.486 [0.115]	84 [30]
		Within 1	25 [7]	30 [22]	0 [0]	0.451 [0.197]	1.000 [0.000]	0.620 [0.185]	
		Between	45 [9]	137 [59]	4 [5]	0.247 [0.082]	0.914 [0.091]	0.389 [0.096]	
		Within 2	24 [6]	29 [21]	0 [0]	0.456 [0.188]	1.000 [0.000]	0.624 [0.177]	
	1	Overall	95 [11]	225 [99]	4 [5]	0.297 [0.102]	0.954 [0.047]	0.453 [0.114]	75 [25]
		Within 1	25 [7]	30 [22]	0 [0]	0.447 [0.191]	1.000 [0.000]	0.618 [0.181]	
		Between	45 [9]	165 [69]	4 [5]	0.218 [0.074]	0.914 [0.087]	0.350 [0.094]	
		Within 2	24 [6]	29 [22]	0 [0]	0.453 [0.187]	1.000 [0.000]	0.622 [0.176]	

Supplementary Table S4: Median and inter-quartile range of the selection performance metrics and computation times obtained with different stability selection models on simulated data with a known block structure. We compare stability selection not accounting for the block structure (Equation (1), denoted by S-B), using block-specific parameters (Equation (4), M-P) and combining block-specific models calibrated while using different penalties λ_0 for the other blocks (Equation (5), M-B). For each block, 30 different penalty parameter values are explored. Models are applied on 1,000 simulated datasets with $p = 100$ variables following a multivariate Normal distribution corresponding to a random graph ($\nu = 0.02$) and with known block structure (50 variables per group, using $v_b = 0.2$). Performances are evaluated in low dimension ($n = 2p = 200$).
